# Supplementary material for: Allosteric regulation and crystallographic fragment screening of SARS-CoV-2 NSP15 endoribonuclease
Source: Nucleic Acids Res. 2023 Apr 28;51(10):5255–70. doi: 10.1093/nar/gkad314 (PMC10250223; doi:10.1093/nar/gkad314)
Supplement: gkad314_Supplemental_Files [file gkad314_supplemental_files.zip › Supplementary datasets.zip!/217/Refine_9/01-BUSTER/Cycle-5/LIST.html]

Refining

**BUSTER 2.X**: A suite of programs for macromolecular structure refinement by maximum-likelihood and Bayesian methods  
Copyright © 1992-2020 Global Phasing Ltd.  
All rights reserved.  
Authors: G. Bricogne, E. Blanc, M. Brandl, C. Flensburg, P. Keller, W. Paciorek, P. Roversi, O. Smart, C. Vonrhein, T. Womack  

Please reference use of the software as:

- BUSTER-TNT 2.X, Global Phasing Ltd, Sheraton House, Cambridge CB3 0AX, UK

Version: 2.10.3 for linux64-ifort made on May 20 2020 at 01:14:34  
Licenced to: Diamond Light Source, Scientific Software  

```
---


### Parsing of the BUSTER cardfile

TITLE : autoBUSTER refinement

   Cell information

   Symmetry information

 Using CCP4 X-ray form factors.

   Missing structure chemical composition

 Using ATOM block for chemical composition.


   Shell information

### Parsing of the TNT cardfile


---


### Reading observations file

 Number of reflexions in input:  47009

      Number of accepted reflexions:  42835

      Number of rejected reflexions:      0

   Preparing complete.mtz containing:      46979 reflexions


   Reflexions in test set:            2171

   Reflexions in working set:        40664


---


### 1. Generating initial model

 2.10 * Shannon sampling rate. NX NY NZ:   288  288  192


  Effective Shannon rates are:    2.44    2.44    2.23


   Allocated BUFFER of size:     61.172 Mb.

   Initial Geometry Information


 No missing atoms declared


   Summary of model volumes

 No missing atoms declared: no prior computed


 Computing bulk solvent model scattering


 NCSMASK PDB radius for solvent mask:     2.15 Angstrom

 running PDBSET (setting cell and spacegroup P1) ... done
 running NCSMASK [1] (creating mask, SMOOTH 1.14715) ... done
 running MAPDUMP (to check if reduction to unit cell is needed) ... done
 running MAPMASK (converting mask to map) ... done

   Generation of babslv by theta-filter

   Electron density in frg mask:  0.420 Å-1

 FOBS  put on rough absolute scale by Wilson plot :
           K        =  0.72616E+02
           B        =     -5.078

 

### Cycle 0001: MaxLik scaling and refinement


   MaxLik Scaling Cycle 0001

   Wilson plot

   Statistics in resolution bins just after scaling
```

|  |  |
| --- | --- |
|  | - Rxpct factors plot- Rxpct factors table- <Log-Lik Gain>- Ln Sigma A- Correlation Coefficients |

```
   <Log-Likelihood> of null-hypothesis

Structure Factor Amplitudes


   Average Figure of Merit

 Switching off refinement of overall temperature factor


 Added B =     0.0269 Å2 to all atoms in fragment


 Added B =     0.0269 Å2 to B_SOLV

```
gelly refinement module
 Authors (2004-2009) O. S. Smart, C. Flensburg, W. Paciorek, C. Vonrhein, 
                     M. Brandl, T. O. Womack and G. Bricogne
         (2010-2011) O. S. Smart, C. Flensburg, W. Paciorek, C. Vonrhein, 
                     T. O. Womack and G. Bricogne
         (2012-2014) O. S. Smart, C. Flensburg, W. Paciorek, C. Vonrhein, 
                     and G. Bricogne
         (2015-2020) G. Bricogne, C. Flensburg, W. Paciorek, C. Vonrhein
 Copyright © 2004-2020 Global Phasing Ltd. 
 All rights reserved.
 gelly module compilation date May 20 2020 01:11:20
 
 gelly uses routines from TNT geometry. References: 
     Tronrud, D. E., Ten Eyck, L. F., Matthews, B. W. (1987)
     "An Efficient General-Purpose Least-Squares Refinement Program 
     for Macromolecular Structures". Acta Crystallogr A, 43:489-501.
     Tronrud, D. E. (1997). "The TNT Refinement Package", 
     in Macromolecular Crystallography, Part B, Eds Charlie Carter 
     and Robert Sweet, Methods in Enzymology, 277:306-319.
 wildcard matching function by Julian Robichaux http://www.nsftools.com

 No -random_seed flag found. Random number stream initialised using:
2147483562
2147483398

 Control flags set to:  -autoncs -hydrogens_input_occ2zero \
                     -hydrogens_output_occ2heavy -screen \
                     100 -screen_sigma \
                     3.0 -keeppdb \
                     1000000 -taomonitor_verbose \
                     2 
 

 ############### Starting setup via TNT routines

 Setting up tnt routines including license check:

                   TNT Stereochemistry Program
 Version: 5.22.3.0
 Licence for Diamond Light Source, Scientific Software is valid
 
 Using TNT geometry routines to load the tnt control file: gelly.tnt
 Using TNT routines to load initial coordinates from file fragment.cor
 Expecting 6517 atoms

 Found     2191 "NOTE BUSTER_RESIDUE_ATOM_DATA" cards
 Have copied information for    6517 atoms from TNT to gelly's store 
 
 The TNT WEIGHT cards used by this run:
 -----WEIGHT       BOND     2.0
 -----WEIGHT       ANGLE    2.0
 -----WEIGHT       IMPROPER 0.0
 -----WEIGHT       TORSION  2.0
 -----WEIGHT       PSEUDO   0.0
 -----WEIGHT       TRIGONAL 2.0
 -----WEIGHT       PLANE    5.0
 -----WEIGHT       CONTACT  5.0
 -----WEIGHT       BCORREL  10.0
 -----WEIGHT       CHIRAL   5.0
 -----WEIGHT       NCS      50.0
 -----WEIGHT       IDEAL    4.0
 -----WEIGHT       RFACTOR  9.64
 
 Dictionary versions picked up from NOTE BUSTER_RESTRAINT_DICT cards as: 
 ------ protgeo_eh99.dat (V1.6.4.17) 20191001  STANDARD AMINO ACID DICTIONARY. BONDS AND ANGLES FROM ENGH AND HUBER EH99. OTHER VALUES BASED ON PREVIOUS TNT OR TAKEN FROM CCP4. INCLUDES HYDROGEN ATOMS.
 ------ exoticaa.dat (V1.3.4.15) 20200327  COLLECTION OF NON-STANDARD AMINO ACIDS, MAINLY EH91 WITHOUT IDEAL DISTANCE INFO
 ------ nuclgeo.dat (V1.13.4.8) 20190909
 ------ bcorrel.dat (V1.15.10.2) 20190616
 ------ contact.dat (V1.15.12.10) 20191121
 ------ idealdist_contact.dat (V1.3.4.12) 20191001  IDEAL-DISTANCE CONTACT TERM DATA AS USED IN PROLSQ. VALUES USED HERE ARE BASED ON DATA HARVESTING OF PDB AS OF 20190109.
 ------ restraints for LIG (Unknown) from cif dictionary Z1269638430.cif
 ------ restraints for CIT (CITRIC ACID) from cif dictionary CIT.cif; generated by GRADE_PDB_LIGAND 1.2.12 (October 4 2016) using MOGUL 1.7.2(RC1), CSD as538be, with quantum mechanics RM1 ((DatabaseID PDB-CIT))
 ------ assume.dat (V1.9.4.2) 20180705
 
 Have picked up spacegroup name for pdb output as 'P 63      '
 Setting up Chain/Residue arrays
 
 Have command line specification -autoncs*
     Have found       1 NCS relations
     Pairwise matching residue number and type between chains: 
                    A        B        S        W    
          A         .      100%       -        -   
          B                  .        -        -   
          S                           .        -   
          W                                    .   
       Table key--                                                       
          all   : two chains have 100% match and same number of residues 
         multi  : ditto and they contribute to a multi chain ncs cluster 
          ***%  : two chains have >80% matching residue number/type      
           -    : chains appear to be unrelated                          
      chain A     have identified ncs to     1 other chains. So will weight each autoncs by (       6.666/#)=      6.6660
      chain B     have identified ncs to     1 other chains. So will weight each autoncs by (       6.666/#)=      6.6660
      chain S     have identified ncs to     0 other chains. 
      chain W     have identified ncs to     0 other chains. 
     For .Gelly cards produced see below - after pruning
 
 Setting up BUSTER_SET groups
 Have found    89 BUSTER_SET cards
 Now silently applying automatic/dictionary set definitions. For a verbose listing use '-verbose_set' flag.
 Applying user defined set definitions:
 =====NOTE BUSTER_SET ncsautoXcld = Water --> set 'ncsautoXcld' now has   966 atoms 
 =====NOTE BUSTER_SET ncsautoABset = Chain_A \ ncsautoXcld --> set 'ncsautoABset' now has  2781 atoms 
 Have defined    49 sets: 
      set:                 'All' has   6517 atoms in   1667 residues - automatically defined
      set:               'Empty' has      0 atoms in      0 residues - automatically defined
      set:            'ExoticAA' has      0 atoms in      0 residues - defined by dictionary file
      set:           'ExoticNUC' has      0 atoms in      0 residues - defined by dictionary file
      set:              'NotHET' has   5514 atoms in    698 residues - defined by dictionary file
      set:             'Chain_A' has   2781 atoms in    351 residues - automatically defined
      set:             'Chain_B' has   2770 atoms in    350 residues - automatically defined
      set:             'Chain_S' has    567 atoms in    567 residues - automatically defined
      set:             'Chain_W' has    399 atoms in    399 residues - automatically defined
      set:               'Water' has    966 atoms in    966 residues - defined by dictionary file
      set:          'StdProtein' has   5514 atoms in    698 residues - defined by dictionary file
      set:             'Protein' has   5514 atoms in    698 residues - defined by dictionary file
      set:                'Back' has   2794 atoms in    698 residues - defined by dictionary file
      set:                'Side' has   2720 atoms in    650 residues - defined by dictionary file
      set:        'RnaCanonical' has      0 atoms in      0 residues - defined by dictionary file
      set:        'DnaCanonical' has      0 atoms in      0 residues - defined by dictionary file
      set:     'RnaDnaCanonical' has      0 atoms in      0 residues - defined by dictionary file
      set:     'DnaRnaCanonical' has      0 atoms in      0 residues - defined by dictionary file
      set:         'RnaExtended' has      0 atoms in      0 residues - defined by dictionary file
      set:         'DnaExtended' has      0 atoms in      0 residues - defined by dictionary file
      set:      'RnaDnaExtended' has      0 atoms in      0 residues - defined by dictionary file
      set:      'DnaRnaExtended' has      0 atoms in      0 residues - defined by dictionary file
      set:                 'Dna' has      0 atoms in      0 residues - defined by dictionary file
      set:                 'Rna' has      0 atoms in      0 residues - defined by dictionary file
      set:              'RnaDna' has      0 atoms in      0 residues - defined by dictionary file
      set:              'DnaRna' has      0 atoms in      0 residues - defined by dictionary file
      set:             'Nucleic' has      0 atoms in      0 residues - defined by dictionary file
      set:             'Polymer' has   5514 atoms in    698 residues - defined by dictionary file
      set: 'BufferWeichenberger' has     26 atoms in      2 residues - defined by dictionary file
      set:    'BufferAdditional' has      0 atoms in      0 residues - defined by dictionary file
      set:              'Buffer' has     26 atoms in      2 residues - defined by dictionary file
      set:         'MetalAlkali' has      0 atoms in      0 residues - defined by dictionary file
      set:  'MetalAlkalineEarth' has      0 atoms in      0 residues - defined by dictionary file
      set:     'MetalTransition' has      0 atoms in      0 residues - defined by dictionary file
      set: 'MetalPostTransition' has      0 atoms in      0 residues - defined by dictionary file
      set:    'MetalLanthanides' has      0 atoms in      0 residues - defined by dictionary file
      set:               'Metal' has      0 atoms in      0 residues - defined by dictionary file
      set:         'SugarAlphaD' has      0 atoms in      0 residues - defined by dictionary file
      set:         'SugarAlphaL' has      0 atoms in      0 residues - defined by dictionary file
      set:               'Sugar' has      0 atoms in      0 residues - defined by dictionary file
      set:               'Other' has     37 atoms in      3 residues - defined by dictionary file
      set:            'Hydrogen' has      0 atoms in      0 residues - defined by dictionary file
      set:         'nonHydrogen' has   6517 atoms in   1667 residues - defined by dictionary file
      set:             'OccZero' has      0 atoms in      0 residues - defined by dictionary file
      set:          'OccNonZero' has   6517 atoms in   1667 residues - defined by dictionary file
      set:             'OccFull' has   6474 atoms in   1664 residues - defined by dictionary file
      set:          'OccNonFull' has     43 atoms in      3 residues - defined by dictionary file
      set:         'ncsautoXcld' has    966 atoms in    966 residues - user specified
      set:        'ncsautoABset' has   2781 atoms in    351 residues - user specified
 No command line arguments '-occ_allhet' or '-occ_restyp' so will not setup automated occcupancy refinement
 
 Interpreting CONSTANT and FREE cards: 
 =====CONSTANT     OCC
 Have found and interpreted      1 CONSTANT cards in the TNT input. 
 Before any COMBINE - Number of atoms with fixed XYZ =     0
 Before any COMBINE - Number of atoms with fixed B   =     0
 Before any COMBINE - Number of atoms with fixed OCC =  6517
 Before any COMBINE - Number of atoms with fixed BIJ =   966
                            cf total number of atoms =  6517
 
 There are no COMBINE cards to be found in the TNT card input 
 
 There are no NOTE BUSTER_COMBINE_RES cards to be found in the TNT card input 

 ############### Working out full connectivity list


 ############### Working out equivalent atoms


 Residue with most atoms given below:
        A|58:N(TRP)         
        A|58:CA(TRP)        
        A|58:CB(TRP)        
        A|58:CG(TRP)        
        A|58:CD1(TRP)       
        A|58:NE1(TRP)       
        A|58:CE2(TRP)       
        A|58:CD2(TRP)       
        A|58:CE3(TRP)       
        A|58:CZ3(TRP)       
        A|58:CH2(TRP)       
        A|58:CZ2(TRP)       
        A|58:C(TRP)         
        A|58:O(TRP)         


 Have no '-equiv_check_plus' argument - so will take atom type into
 account when checking for equivalence of otherwise identical atoms.
 

 ############### Looking at dihedrals


 ############### Looking at chirals

 NOTE BUSTER_CHIRAL_ANGLECUT card found - read value=    -31.00 degs from it
 NOTE BUSTER_CHIRAL_SIGMA card found - read value=      1.70 degs from it
 RANGE allowed for B variables in refinement:   from      3.00000 to    300.00000

 ############### Preparing for PDB output


 ############### Processing extra restraints explicitly defined

 No 'NOTE BUSTER_TARGET' cards or -target?? args found (used to read coordinates files for soft ncs to external)
 No 'NOTE BUSTER_OCCSUM'     cards found - so not using sum of occupancy restraints
 No 'NOTE BUSTER_DISTANCE'   cards found - so not setting up any additional atom-atom distance restraints
 No 'NOTE BUSTER_UTILANGLE'  cards found - so not setting up any additional restraints on any angle
 No 'NOTE BUSTER_UTILTOR'    cards found - so not setting up any additional sinusoidal restraints on torsions
 
 --- Have some similarity definitions 
     Similarity restraints on RMSD use superposition routines from: 
     Coutsias, E.A., Seok, C., Dill, K.A.(2004)
     "Using quaternions to calculate RMSD", J. Comput. Chem., 25:1849-1857 

 Have found    1 'NOTE BUSTER_SIM_DEFINE' cards 
     Maximum number of chains involved in any SIM group (excluding the template)=     1


 ########## Interpreting card: NOTE BUSTER_SIM_DEFINE ncsautoAB ncsautoABset B
     Number of atoms in template set=  2781
     Number of related chains (other than template) in SIM set=    1
     List of chains to be restrained (excluding template)=   B   
     Pairwise RMSD, XYZ in Angstroms, for SIM group: ncsautoAB
                 A         B   
     A         -----     ----- 
     B         0.642     ----- 
     Overall RMSD,XYZ for the SIM group is     0.642 Angstroms
     Pairwise rms deviation of [individual B's minus their set/chain average] in Angs**2
                 A         B         Bavg
     A         -----     -----     97.16
     B         38.80     -----     69.06
     Overall RMSD,B   for the SIM group is    38.803 Angs**2
     The number of common atoms for the above fits:
                 A         B   
     A         -----     ----- 
     B          2770     ----- 
     (compared to the template set that has    2781 atoms in it)
     List of atoms that are absent in one or more chains for this group: 
                                    A    B   
                    C01     501     +    -
                    C02     501     +    -
                    O03     501     +    -
                    N04     501     +    -
                    C05     501     +    -
                    C06     501     +    -
                    C07     501     +    -
                    N08     501     +    -
                    C09     501     +    -
                    C10     501     +    -
                    O11     501     +    -
     (key  +  atom present for this chain 
           -  atom absent  for this chain)
 transforms for each of the superpositions orthogonal matrix r11 r12 r13 r21 r22 r23 r31 r32 r33 tx ty tz (PDBSET syntax): 
TRANS_AB -0.872160 -0.488165 -0.032114 -0.488107  0.872725 -0.010151  0.032982  0.006822 -0.999433    33.873    33.085    14.691
 reverse transforms 
TRANS_BA -0.872160 -0.488107  0.032982 -0.488165  0.872725  0.006822 -0.032114 -0.010151 -0.999433    45.207   -12.439    16.106
 
 Setting up LSSR interatomic distance restraints
 Interpreting card: NOTE BUSTER_SIM_RESTRAIN_LSSR ncsautoAB 6.666
        setting up interatomic distance pair restraints with:
        Dmin=         0.00
        Dmax=         5.50
        sigma=        0.20
        Vmax=         3.00
        weight=       6.67
        number of distance pairs defined for this set is      35089
 Have not supplied either '-sim_swap_equiv' or '-sim_swap_equiv_plus' argument 
   so will not try swaping equivalent atoms to improve NCS and/or target agreement.
 
 NCS by LSSR outlier analysis
 -autoncs option automatic NCS prune routine -------- 
 Residues pruned out by gradient condition:
           A|23      A|27      A|30      A|33      A|34      A|54      A|87
          A|135     A|138     A|139     A|148     A|149     A|150     A|151
          A|152     A|181     A|182     A|183     A|184     A|185     A|186
          A|187     A|189     A|216     A|217     A|219     A|220     A|250
          A|253     A|255     A|259     A|264     A|319     A|328     A|344
          A|345      B|23      B|27      B|30      B|33      B|34      B|54
           B|87     B|135     B|138     B|139     B|148     B|151     B|152
          B|181     B|182     B|183     B|184     B|185     B|186     B|187
          B|189     B|216     B|217     B|219     B|220     B|250     B|253
          B|255     B|259     B|264     B|319     B|328     B|344     B|345
       
 Residues pruned out by function/max condition:
          A|149!    A|187!    A|188     A|264!    A|328!    A|346    A|ENDA 
          A|401     B|149     B|187!    B|188     B|264!    B|328!    B|346 
         B|ENDA     B|401 
  
 Total number of residues for which LSSR restraints have been pruned out is       79 out of      700 participating
 Number of LSSR restraints pruned out is      5665 this is   16.1% of the total           35089
  
 -autoncs .Gelly cards suitable for adaptation for manual definition/pruning
           (note auto pruning is done in a slightly different way so exact results may differ slightly)
 -autoncs option .Gelly cards also written to file  auto_gelly_cards.txt
NOTE BUSTER_SET ncsautoXcld = Water    # card added - produced by -autoncs
NOTE BUSTER_SET ncsautoXcld = ncsautoXcld + { A|23 A|27 A|30 A|33 A|34 A|54 }    # card added - produced by -autoncs
NOTE BUSTER_SET ncsautoXcld = ncsautoXcld + { A|87 A|135 A|138 A|139 A|148 }    # card added - produced by -autoncs
NOTE BUSTER_SET ncsautoXcld = ncsautoXcld + { A|149 A|150 A|151 A|152 A|181 }    # card added - produced by -autoncs
NOTE BUSTER_SET ncsautoXcld = ncsautoXcld + { A|182 A|183 A|184 A|185 A|186 }    # card added - produced by -autoncs
NOTE BUSTER_SET ncsautoXcld = ncsautoXcld + { A|187 A|188 A|189 A|216 A|217 }    # card added - produced by -autoncs
NOTE BUSTER_SET ncsautoXcld = ncsautoXcld + { A|219 A|220 A|250 A|253 A|255 }    # card added - produced by -autoncs
NOTE BUSTER_SET ncsautoXcld = ncsautoXcld + { A|259 A|264 A|319 A|328 A|344 }    # card added - produced by -autoncs
NOTE BUSTER_SET ncsautoXcld = ncsautoXcld + { A|345 A|346 A|ENDA A|401 }    # card added - produced by -autoncs
NOTE BUSTER_SET ncsautoABset = Chain_A \ ncsautoXcld    # card added - produced by -autoncs
NOTE BUSTER_SIM_DEFINE ncsautoAB ncsautoABset B    # card added - produced by -autoncs
NOTE BUSTER_SIM_RESTRAIN_LSSR ncsautoAB 6.666    # card added - produced by -autoncs

 LSSR function contribution before pruning     47660.5
                             after pruning     11417.8
 Graph of which residues pruned from LSSR: ncs_pruned.mtv
 Graph of LSSR function outliers ncs_function_outliers.mtv
 Graph of LSSR gradient outliers ncs_gradient_outliers.mtv
 LSSR - analysis of distance difference distribution for NCS
         DeltaD less than 0.5 Angs:  proportion of population= 98.64% average DeltaD of subset     0.015 Angs
 Histogram of LSSR deltaD distribution ncs_histogram.mtv
 
 Interpreting card: NOTE BUSTER_TLS_SET tls1 { A|* }
 Interpreting card: NOTE BUSTER_TLS_SET tls2 { B|* }
 Interpreting card: NOTE BUSTER_TLS_SET tls1 { A|* }
 Interpreting card: NOTE BUSTER_TLS_T   tls1     -9.84122E-02  2.63375E-02  4.22310E-02 -5.87256E-02  1.84806E-02 -4.63797E-02
 Interpreting card: NOTE BUSTER_TLS_L   tls1      2.53184E+00  2.05865E+00  1.86779E+00 -8.79892E-01 -1.59062E+00  7.18718E-01
 Interpreting card: NOTE BUSTER_TLS_S   tls1     -1.17821E-02 -3.86196E-01  5.27491E-01 -4.35968E-01  2.67147E-01 -2.69276E-01  6.09961E-02 -3.35657E-01
 Interpreting card: NOTE BUSTER_TLS_O   tls1     -2.30832E+01 -8.41146E+01  1.12764E+01
 Interpreting card: NOTE BUSTER_TLS_FIX tls1 ALL
 Interpreting card: NOTE BUSTER_TLS_SET tls2 { B|* }
 Interpreting card: NOTE BUSTER_TLS_T   tls2     -1.73365E-01 -1.10956E-01  1.17182E-01  1.49139E-02  2.71700E-02 -2.71783E-02
 Interpreting card: NOTE BUSTER_TLS_L   tls2      1.57079E+00  1.30451E+00  1.13623E+00  8.10314E-01 -1.11889E+00 -8.20951E-01
 Interpreting card: NOTE BUSTER_TLS_S   tls2     -7.19371E-02  1.20184E-01 -9.00838E-02  1.24667E-02  1.65761E-01  7.66243E-02 -1.29102E-01  7.22209E-02
 Interpreting card: NOTE BUSTER_TLS_O   tls2      2.22817E+01 -7.23258E+01  3.51644E+00
 Interpreting card: NOTE BUSTER_TLS_FIX tls2 ALL


 TLS information. Total number of groups:     2


 Group   Natoms    FixRB   FixTLS   Group Tag    Group Spec
     1     2781      Y        Y     tls1    { A|* }
     2     2770      Y        Y     tls2    { B|* }
 
NOTE BUSTER_TLS_SET tls1 { A|* }
NOTE BUSTER_TLS_T   tls1     -9.84122E-02  2.63375E-02  4.22310E-02 -5.87256E-02  1.84806E-02 -4.63797E-02
NOTE BUSTER_TLS_L   tls1      2.53184E+00  2.05865E+00  1.86779E+00 -8.79892E-01 -1.59062E+00  7.18718E-01
NOTE BUSTER_TLS_S   tls1     -1.17821E-02 -3.86196E-01  5.27491E-01 -4.35968E-01  2.67147E-01 -2.69276E-01  6.09961E-02 -3.35657E-01
NOTE BUSTER_TLS_O   tls1     -2.30832E+01 -8.41146E+01  1.12764E+01
NOTE BUSTER_TLS_FIX tls1 ALL
 
NOTE BUSTER_TLS_SET tls2 { B|* }
NOTE BUSTER_TLS_T   tls2     -1.73365E-01 -1.10956E-01  1.17182E-01  1.49139E-02  2.71700E-02 -2.71783E-02
NOTE BUSTER_TLS_L   tls2      1.57079E+00  1.30451E+00  1.13623E+00  8.10314E-01 -1.11889E+00 -8.20951E-01
NOTE BUSTER_TLS_S   tls2     -7.19371E-02  1.20184E-01 -9.00838E-02  1.24667E-02  1.65761E-01  7.66243E-02 -1.29102E-01  7.22209E-02
NOTE BUSTER_TLS_O   tls2      2.22817E+01 -7.23258E+01  3.51644E+00
NOTE BUSTER_TLS_FIX tls2 ALL


 TLS: mmCIF group specification begin


#
loop_
_pdbx_refine_tls_group.id
_pdbx_refine_tls_group.refine_tls_id
_pdbx_refine_tls_group.beg_PDB_ins_code
_pdbx_refine_tls_group.beg_auth_asym_id
_pdbx_refine_tls_group.beg_auth_seq_id
_pdbx_refine_tls_group.end_PDB_ins_code
_pdbx_refine_tls_group.end_auth_asym_id
_pdbx_refine_tls_group.end_auth_seq_id
_pdbx_refine_tls_group.selection
_pdbx_refine_tls_group.selection_details
   1   1 . A   -1 . A  346 . '{ A|* }'
   2   1 . A  401 . A  501 . '{ A|* }'
   3   2 . B   -1 . B  346 . '{ B|* }'
   4   2 . B  401 . B  401 . '{ B|* }'
#


 TLS: mmCIF group specification end


 Interpreting card: NOTE BUSTER_ANISO_SET Empty
 Interpreting card: NOTE BUSTER_ANISO_WEIGHT SCA  16.0
 Interpreting card: NOTE BUSTER_ANISO_WEIGHT SPH   0.6    0.6
 Interpreting card: NOTE BUSTER_ANISO_WEIGHT SIM   7.8    4.8    2.6    0
 Interpreting card: NOTE BUSTER_ANISO_WEIGHT RIG  21.5   21.5    3.7    0


 After TLS and ANISO: Number of atoms with fixed/combined xyz =     0
 After TLS and ANISO: Number of atoms with fixed/combined B   =     0
 After TLS and ANISO: Number of atoms with fixed/combined OCC =  6517
 After TLS and ANISO: Number of atoms with fixed/combined BIJ =  6517
 After TLS and ANISO: Number of atoms in a TLS group          =  5551
                                     cf total number of atoms =  6517
 

 ############### Setup state vector

 No free_vect_ulim_xyz argument found: using default of  100.000
 No free_vect_llim_xyz argument found: using default of -100.000
 Number of parameters added for TLS description                 =       0
 Number of parameters to describe molecular conformation        =   26068

 ############### Setup free vector

 Number of free parameters to be adjusted in refinement   =   26068

 ############### Setting up own geometry


 No command line argument '-tnt_fn' so will use gelly to do geometry function calculations

 ============= Call TNT functions


 ============= Get TNT symmetry information

 
      Getting symmetry operators from TNT. 
          gelly will classify symmetry using pdb-like convention: 
               SYMOP   SYMMETRY
              NNNMMM   OPERATOR
                1555   X,Y,Z
                2555   -Y,X-Y,Z
                3555   -X+Y,-X,Z
                4555   -X,-Y,1/2+Z
                5555   Y,-X+Y,1/2+Z
                6555   X-Y,X,1/2+Z
          where NNN -> operator number and MMM -> translation vector
 

 ============= Own Gelly geometry/restraints setup


 ----------- Identify bond-types


    Setting up GEOMBONDREC_TYPE_HASH* to be able to map a given bond type (e.g.
    "single") to an internal integer (GBOND_TYPE=1) etc.

    Using default  GEOMBONDREC_TYPE_HASH_STR='SING* 1 DOUB* 2 TRIP* 3 AROM* 4 DELO* 4 METAL* 5':
    to override default use a NOTE BUSTER_GEOMBONDREC_TYPE_HASH_STR gelly card.

 ----------- Classify torsion into peptide omega and other


 ----------- Setup of non-bonded terms

 Ideal-distance contact term setup:
    Restraint function constants (normally these values are from NOTE BUSTER_IDEAL_CONTACT_* cards)
    Sigma for general contacts      0.200 Angs
    Sigma for 1-4 contacts          0.100 Angs (contacts across a torsion)
    Sigma for 1-5 contacts          0.150 Angs
    Sigma for 1-N contacts          0.200 Angs
    Sigma for HBOND pairs           0.100 Angs (contacts between HB_types A and D or B's)
    Contact distance increment for each atom in HBOND          -0.150 Angs 
    Contact distance increment for H-bond acceptor              0.100 Angs 
    Contact distance increment for 1-4 with an O atom          -0.050 Angs 
    Contact distance increment for 1-4 with an N atom          -0.180 Angs 
    Contact distance increment for 1-4 with an C atom          -0.370 Angs 
    Contact distance increment for 1-4 with an S atom          -0.150 Angs 
    Contact distance increment for 1-4 with an P atom          -0.150 Angs 
    Contact distance increment for 1-4 with any other atom     -0.150 Angs 
    All atoms matched for CCP4 atom type.
    Number of CCP4 atom types used is         24 (H-bound and not H-bound counted separately)

 Setting up short/ideal-distance contacts
   Using gelly special position handling. (For TNT instead rerun with argument '-special_tnt')

   No command line argument '-special_all' specified. We will auto-generate
   EXCLUDE cards only for atom-atom contacts across symmetry (i.e. on special
   position) when this involves a contact within a residue to itself.

   Have set up non-bonded exclusions - 1-3 contacts
   Atom A|27:CA         makes the most 1-3 contacts:  6

   Have set up non-bonded exclusions - 1-4 contacts
   Atom A|27:N          makes the most 1-4 contacts:  7

   Have set up non-bonded exclusions - 1-5 contacts
   Atom A|28:C.A        makes the most 1-5 contacts: 10

 ############################################################################################################
 ###################################################### setting up contact terms
 ############################################################################################################


 ============= Call Gelly versions of TNT functions

 WARNING: 1-4 contact without 1-(2,3)-4 data for A|401:C2(CIT)        and A|401:O6(CIT)        X-X
 WARNING: 1-4 contact without 1-(2,3)-4 data for A|401:O7(CIT)        and A|401:C5(CIT)        X-X
 WARNING: 1-4 contact without 1-(2,3)-4 data for A|401:O7(CIT)        and A|401:O5(CIT)        X-X
 WARNING: 1-4 contact without 1-(2,3)-4 data for A|401:C4(CIT)        and A|401:O6(CIT)        X-X
 WARNING: 1-4 contact without 1-(2,3)-4 data for A|501:C01(LIG)       and A|501:C05(LIG)       X-X
 WARNING: 1-4 contact without 1-(2,3)-4 data for A|501:C06(LIG)       and A|501:C09(LIG)       X-X
 WARNING: 1-4 contact without 1-(2,3)-4 data for A|501:C07(LIG)       and A|501:C10(LIG)       X-X
 WARNING: 1-4 contact without 1-(2,3)-4 data for B|401:C1(CIT)        and B|401:C4(CIT)        X-X
 WARNING: 1-4 contact without 1-(2,3)-4 data for B|401:C2(CIT)        and B|401:O6(CIT)        X-X
 WARNING: 1-4 contact without 1-(2,3)-4 data for B|401:O7(CIT)        and B|401:O5(CIT)        X-X
 WARNING: 1-4 contact without 1-(2,3)-4 data for B|401:C4(CIT)        and B|401:O6(CIT)        X-X

       TERM      weight      number      TNT_fn    Gelly_fn        diff
       BOND       2.000        5665    1977.149    1977.149       0.000
       ANGL       2.000        7687    5768.588    5768.588       0.000
       IMPR       0.000           0       0.000       0.000       0.000
       TORS       2.000        1973    3525.341    3525.341      -0.000
       TRIG       2.000           0       0.000       0.000       0.000
       PLAN       5.000         967    2656.379    2656.379       0.000
       CONT       5.000           0     122.164       0.000     122.164
       BCOR      10.000        5630   10582.128   10030.947     551.182


 ============= Call Gelly geometry functions

   Switching torsion functional form to sinusoidal rather than split harmonic functional.
      if you want this not to happen then rerun with '-torsharm'
      Weight for sinusoidal torsion=     2.000
   BCOR function/gradient will be split between H and non-H atoms (as for any atom pair). Use '-bcorrel_all_in_hydrogens' to change this.

 ----------- Fix B-correlation restraints

 
 Checking for bonds that do not have a bcorrel restraint defined
 We will wipe all existing BCORrels if OCC=0.0 atom(s) is/are involved. This can be' switched off via the '-bcorrel_nowipe_zeroocc' flag.
 We will wipe all existing BCORrels if only non-hydrogen atom(s) with fixed B(iso) are involved. This can be switched off via the '-bcorrel_nowipe_fixb_nonhydrogens' flag.
 We will keep all existing BCORrels if hydrogen atom(s) with fixed B(iso) is/are involved. This can be switched off via the '-bcorrel_wipe_fixb_hydrogens' flag.
 No -bcorrel_missing_sigma found. So will set sigma to     2.5 Ang**2 for any bond with no existing BCORrel
 No -bcorrel_missing_sigma_hydrogens found. So will set sigma to     1.0 Ang**2 for any bond with no existing BCORrel involving hydrogens

 No active BCORels found involving OCC=0.0 atom(s)


 No active BCORels found involving fixed-B (only) non-hydrogen atom(s)

    add a default BCOR for bond A|401:C1(CIT) A|401:C2(CIT)
    add a default BCOR for bond A|401:C1(CIT) A|401:O1(CIT)
    add a default BCOR for bond A|401:C1(CIT) A|401:O2(CIT)
    add a default BCOR for bond A|401:C2(CIT) A|401:C3(CIT)
    add a default BCOR for bond A|401:C3(CIT) A|401:C4(CIT)
    add a default BCOR for bond A|401:C3(CIT) A|401:C6(CIT)
    add a default BCOR for bond A|401:C3(CIT) A|401:O7(CIT)
    add a default BCOR for bond A|401:C4(CIT) A|401:C5(CIT)
    add a default BCOR for bond A|401:C5(CIT) A|401:O3(CIT)
    add a default BCOR for bond A|401:C5(CIT) A|401:O4(CIT)
    add a default BCOR for bond A|401:C6(CIT) A|401:O5(CIT)
    add a default BCOR for bond A|401:C6(CIT) A|401:O6(CIT)
    add a default BCOR for bond A|501:C01(LIG) A|501:C02(LIG)
    add a default BCOR for bond A|501:C02(LIG) A|501:O03(LIG)
    add a default BCOR for bond A|501:C02(LIG) A|501:N04(LIG)
    add a default BCOR for bond A|501:N04(LIG) A|501:C05(LIG)
    add a default BCOR for bond A|501:C05(LIG) A|501:C06(LIG)
    add a default BCOR for bond A|501:C06(LIG) A|501:C07(LIG)
    add a default BCOR for bond A|501:C06(LIG) A|501:O11(LIG)
    add a default BCOR for bond A|501:C07(LIG) A|501:N08(LIG)
    add a default BCOR for bond A|501:N08(LIG) A|501:C09(LIG)
    add a default BCOR for bond A|501:C09(LIG) A|501:C10(LIG)
    add a default BCOR for bond A|501:C10(LIG) A|501:O11(LIG)
    add a default BCOR for bond B|401:C1(CIT) B|401:C2(CIT)
    add a default BCOR for bond B|401:C1(CIT) B|401:O1(CIT)
    add a default BCOR for bond B|401:C1(CIT) B|401:O2(CIT)
    add a default BCOR for bond B|401:C2(CIT) B|401:C3(CIT)
    add a default BCOR for bond B|401:C3(CIT) B|401:C4(CIT)
    add a default BCOR for bond B|401:C3(CIT) B|401:C6(CIT)
    add a default BCOR for bond B|401:C3(CIT) B|401:O7(CIT)
    add a default BCOR for bond B|401:C4(CIT) B|401:C5(CIT)
    add a default BCOR for bond B|401:C5(CIT) B|401:O3(CIT)
    add a default BCOR for bond B|401:C5(CIT) B|401:O4(CIT)
    add a default BCOR for bond B|401:C6(CIT) B|401:O5(CIT)
    add a default BCOR for bond B|401:C6(CIT) B|401:O6(CIT)

 Have added a total of        35 BCORrels for bonds which do not already have a BCORrel


 No bonds (for potential BCORrel addition) were skipped due to both atoms having constant/fixed B(iso)


 ----------- Deactivate hydrogen-free planes if hydrogen atoms present

 A total of       0 planes have been deactivated because they are duplicated hydrogen-free and with-hydrogen forms.

 ----------- Handle peptide omega angle(s)

      Torsion angles split into peptide omega and other
         Number of peptide omega angles is       698
         Number of other torsion is             1275
      Peptide omega angles are monitored rather than directly restrained (PLANE used instead).

 ----------- Fix cis-PRO

 Now apply the fix to get correct restraints for cis PRO residues
   Have got      11 bonds records and      26 angles
   from GEOMETRY cards for the special PROC residue
   Weight for ideal-distance contact term=     4.000

 ----------- Prepare SSBOND/LINK for output PDB file


 ----------- Prepare for QM


 ----------- Prepare symmetry in utility distance restraints


 ############### Setting up Rhofit contacts (if needed)

 No ligand-(fixed protein) contact term will be used

 ############### Working out initial function and gradient


   MaxLik Scaling Cycle 0002
 
 Initial functional value = 4.771862E+05 rms gradient=      46.1847
 X-ray contribution to function value = 4.293489E+05
 Geometry contribution (inc ncs)      =      47837.3
 Geometry function summary
     term                           number     weight       rms        GooF      function     fn/numb
     ================================================================================================
     BOND bond lengths (angs)       5665       2.000      0.0084       0.418      1977.15       0.349
     ANGL bond angles (degs)        7687       2.000        1.03       0.613      5768.59       0.750
     TORS split-harmonic tors       1275       0.000       18.70       1.176         0.00       0.000
     SINTOR sinusoidal tors         1275       2.000       18.70                  2601.94       2.041
     PLAN general plane (angs)       967       5.000      0.0148       0.741      2656.38       2.747
     BCORrelations (angs^2)         5665      10.000       1.179       0.421     10032.17       1.771
     CONT (bad contacts)               0       5.000                                 0.00
     IDEAL(ideal-dist contact)      5767       4.000                             13327.25
     CHIRAL (gelly semiharmon)       740       5.000                                56.05
     SIM similarity restraint for NCS and/or TARGET                              11417.78
     OCCSUM restraint on sum of occupanies                                           0.00       as none defined
     DISTAN utility restraints interatomic distance                                  0.00       as none defined
     UTILANGLE utility angle restraints                                              0.00       as none defined
     UTILTOR utility restraints on torsion angles                                    0.00       as none defined
     Peptide omega torsion angles                           2.98 degs - monitor only

 Weighted rms      0.0084         1.028  (temporary rms bond and angle for BUSTER to pick up!)
 Weighted intra-residual rms(bond) for StdProtein (excluding H)      0.0082
 Weighted intra-residual rms(bond) for Buffer     (excluding H)      0.0035

 ############### Running initial check


 Initial Geometry Sanity Check

 Check for duplicate bonds: no duplicates found
 
 Check for duplicate or missing bond angles: all are OK
 Check for bond between an atom and itself not involving symmetry:  all are OK
   There are no bad contacts where Delta/sigma is greater than     6.5
   Worst contact dist=   2.933 std=   3.802 sigma=   0.206 abs(Delta/sigma)=    4.217 ats A|104:CE (MET) B|23:CB (PRO)
   It is likely that the geometry libraries, sequence info and links match the input molecule
 GEOMETRY CHECK OVERALL:  PASS


 ############### Creating geometry/screen report


 ############### Option "tao" - run Tao optimizer

 TAO: Toolkit for Advanced Optimization. Reference: 
     Munson, T., Sarich, J., Wild, S., Benson, S., McInnes, L.C. (2018)
     "TAO User Manual (Revision 3.9)", Mathematics and Computer Science
     Division, Argonne National Laboratory
     ANL/MCS-TM-322, http://www.mcs.anl.gov/petsc/
 
 Have found -keeppdb flag so will write out a .pdb file every 1000000 its during opt
 Will apply TAO optimization 
 Maximum number of iterations      100
 Convergence test on Grms       4.0000 (-glim)
 Limit on rms displacement to original position (rmsD2init): NONE test inactive (-dlim)

    analysing argument no 0 : -autoncs with j=0
    analysing argument no 1 : -hydrogens_input_occ2zero with j=0
    analysing argument no 2 : -hydrogens_output_occ2heavy with j=0
    analysing argument no 3 : -screen with j=0
    analysing argument no 4 : 100 with j=0
    analysing argument no 5 : -screen_sigma with j=0
    analysing argument no 6 : 3.0 with j=0
    analysing argument no 7 : -keeppdb with j=0
    analysing argument no 8 : 1000000 with j=0
    analysing argument no 9 : -taomonitor_verbose with j=0
    found argument starting with "-tao" : -taomonitor_verbose
    analysing argument no 10 : 2 with j=1
    found value after -tao/-options_/-viewer_ argument : "2"
 PETSC/TAO: setting option "-taomonitor_verbose" = "2"


   MaxLik Scaling Cycle 0003
   Iters     Tot_Funct    Xray_Funct    Geom_Funct          Grms     rmsD2init     maxD2init             R         Rfree           LLG       LLGfree     rms(bond)    rms(angle)       Opttag_H
   Iters             K   B_IMPF_FRAG        K_MISS   B_TEMP_MISS   K_IMPF_MISS   B_IMPF_MISS        K_SOLV   B_TEMP_SOLV   K_IMPF_SOLV   B_IMPF_SOLV        B_TEMP     B_TEMP_11     B_TEMP_12     B_TEMP_22     B_TEMP_13     B_TEMP_23     B_TEMP_33      K_WILSON      B_WILSON      FGCYCLES       Scatag_H
   Iters          BOND         ANGLE      IMPROPER       TORSION    SINTORSION      TRIGONAL        PLANAR  BCORRELATION       CONTACT     IDEALDIST        CHIRAL    SIMILARITY       OCCSSUM  UTILDISTANCE     UTILANGLE   UTILTORSION        RHOFIT        ADP-01        ADP-02        ADP-03        ADP-04        ADP-05        ADP-06        ADP-07        ADP-08        ADP-09        ADP-10      RHOFITPC            QM       Funtag_H
       0  4.771224E+05  4.293551E+05       47767.4       43.3451  0.000000E+00  0.000000E+00      0.184747      0.239430  0.000000E+00  0.000000E+00    0.00837575       1.02787       Opttag_D
       0       83.3923       1.13555       1.00000  0.000000E+00       1.00000  0.000000E+00      0.947775       89.4578       1.00000       37.8287  0.000000E+00       8.89035  0.000000E+00       8.89035  0.000000E+00  0.000000E+00      -17.7807       85.2537       3.90356        2       Scatag_D
       0       1977.15       5768.59  0.000000E+00  0.000000E+00       2601.94  0.000000E+00       2656.38       9962.23  0.000000E+00       13327.2       56.0503       11417.8 -1.000000E+10 -1.000000E+10 -1.000000E+10 -1.000000E+10 -1.000000E+10  0.000000E+00  0.000000E+00  0.000000E+00  0.000000E+00  0.000000E+00  0.000000E+00  0.000000E+00  0.000000E+00  0.000000E+00  0.000000E+00  0.000000E+00  0.000000E+00       Funtag_D
   Geometry Restraint Deviations SCREEN Information

 

 An atom has moved more than     1.50 A from the initial/last
 position - if this was an accepted trial point, probably need to update the
 non-bonded contact list.


   MaxLik Scaling Cycle 0004

   MaxLik Scaling Cycle 0005

   MaxLik Scaling Cycle 0006

   Wilson plot

   Statistics in resolution bins after refinement cycle 0001
```


|  |  |
| --- | --- |
|  | - Rxpct factors plot- Rxpct factors table- <Log-Lik Gain>- Ln Sigma A- Correlation Coefficients |


```
   Iters     Tot_Funct    Xray_Funct    Geom_Funct          Grms     rmsD2init     maxD2init             R         Rfree           LLG       LLGfree     rms(bond)    rms(angle)
   Iters             K   B_IMPF_FRAG        K_MISS   B_TEMP_MISS   K_IMPF_MISS   B_IMPF_MISS        K_SOLV   B_TEMP_SOLV   K_IMPF_SOLV   B_IMPF_SOLV        B_TEMP     B_TEMP_11     B_TEMP_12     B_TEMP_22     B_TEMP_13     B_TEMP_23     B_TEMP_33      K_WILSON      B_WILSON      FGCYCLES
   Iters          BOND         ANGLE      IMPROPER       TORSION    SINTORSION      TRIGONAL        PLANAR  BCORRELATION       CONTACT     IDEALDIST        CHIRAL    SIMILARITY       OCCSSUM  UTILDISTANCE     UTILANGLE   UTILTORSION        RHOFIT        ADP-01        ADP-02        ADP-03        ADP-04        ADP-05        ADP-06        ADP-07        ADP-08        ADP-09        ADP-10      RHOFITPC            QM
       1  4.767203E+05  4.293733E+05       47347.0       33.4168  3.220656E-04    0.00417077      0.184711      0.239632  1.698370E-04 -8.175506E-04    0.00835266       1.02775       Opttag_D
       1       83.3932       1.13619       1.00000  0.000000E+00       1.00000  0.000000E+00      0.947785       89.4514       1.00000       37.8117  0.000000E+00       8.89016  0.000000E+00       8.89016  0.000000E+00  0.000000E+00      -17.7803       85.2537       3.90356        3       Scatag_D
       1       1966.26       5767.25  0.000000E+00  0.000000E+00       2601.85  0.000000E+00       2654.84       9576.08  0.000000E+00       13324.6       55.8401       11400.3 -1.000000E+10 -1.000000E+10 -1.000000E+10 -1.000000E+10 -1.000000E+10  0.000000E+00  0.000000E+00  0.000000E+00  0.000000E+00  0.000000E+00  0.000000E+00  0.000000E+00  0.000000E+00  0.000000E+00  0.000000E+00  0.000000E+00  0.000000E+00       Funtag_D
   Geometry Restraint Deviations SCREEN Information

 

   MaxLik Scaling Cycle 0007

   MaxLik Scaling Cycle 0008

   Wilson plot

   Statistics in resolution bins after refinement cycle 0002
```


|  |  |
| --- | --- |
|  | - Rxpct factors plot- Rxpct factors table- <Log-Lik Gain>- Ln Sigma A- Correlation Coefficients |


```
   Iters     Tot_Funct    Xray_Funct    Geom_Funct          Grms     rmsD2init     maxD2init             R         Rfree           LLG       LLGfree     rms(bond)    rms(angle)
   Iters             K   B_IMPF_FRAG        K_MISS   B_TEMP_MISS   K_IMPF_MISS   B_IMPF_MISS        K_SOLV   B_TEMP_SOLV   K_IMPF_SOLV   B_IMPF_SOLV        B_TEMP     B_TEMP_11     B_TEMP_12     B_TEMP_22     B_TEMP_13     B_TEMP_23     B_TEMP_33      K_WILSON      B_WILSON      FGCYCLES
   Iters          BOND         ANGLE      IMPROPER       TORSION    SINTORSION      TRIGONAL        PLANAR  BCORRELATION       CONTACT     IDEALDIST        CHIRAL    SIMILARITY       OCCSSUM  UTILDISTANCE     UTILANGLE   UTILTORSION        RHOFIT        ADP-01        ADP-02        ADP-03        ADP-04        ADP-05        ADP-06        ADP-07        ADP-08        ADP-09        ADP-10      RHOFITPC            QM
       2  4.765846E+05  4.293894E+05       47195.2       36.7068  7.514517E-04    0.00883639      0.184717      0.239649  1.287226E-04 -8.657940E-04    0.00832980       1.02920       Opttag_D
       2       83.3943       1.13672       1.00000  0.000000E+00       1.00000  0.000000E+00      0.947793       89.4375       1.00000       37.7894  0.000000E+00       8.88949  0.000000E+00       8.88949  0.000000E+00  0.000000E+00      -17.7790       85.2537       3.90356        2       Scatag_D
       2       1955.51       5783.48  0.000000E+00  0.000000E+00       2601.74  0.000000E+00       2652.60       9447.88  0.000000E+00       13319.8       55.7657       11378.4 -1.000000E+10 -1.000000E+10 -1.000000E+10 -1.000000E+10 -1.000000E+10  0.000000E+00  0.000000E+00  0.000000E+00  0.000000E+00  0.000000E+00  0.000000E+00  0.000000E+00  0.000000E+00  0.000000E+00  0.000000E+00  0.000000E+00  0.000000E+00       Funtag_D
   Geometry Restraint Deviations SCREEN Information

 
       3  4.764781E+05  4.294040E+05       47074.1       29.7006    0.00110552     0.0136887      0.184719      0.239663  9.164279E-05 -9.042537E-04    0.00831722       1.02783       Opttag_D
       3       83.3955       1.13723       1.00000  0.000000E+00       1.00000  0.000000E+00      0.947800       89.4283       1.00000       37.7701  0.000000E+00       8.88893  0.000000E+00       8.88893  0.000000E+00  0.000000E+00      -17.7779       85.2537       3.90356        1       Scatag_D
       3       1949.61       5768.12  0.000000E+00  0.000000E+00       2601.59  0.000000E+00       2650.76       9369.35  0.000000E+00       13315.5       55.7438       11363.4 -1.000000E+10 -1.000000E+10 -1.000000E+10 -1.000000E+10 -1.000000E+10  0.000000E+00  0.000000E+00  0.000000E+00  0.000000E+00  0.000000E+00  0.000000E+00  0.000000E+00  0.000000E+00  0.000000E+00  0.000000E+00  0.000000E+00  0.000000E+00       Funtag_D
       4  4.763949E+05  4.294181E+05       46976.8       26.4399    0.00153040     0.0192844      0.184723      0.239682  5.561968E-05 -9.580074E-04    0.00830956       1.02742       Opttag_D
       4       83.3969       1.13776       1.00000  0.000000E+00       1.00000  0.000000E+00      0.947807       89.4189       1.00000       37.7472  0.000000E+00       8.88832  0.000000E+00       8.88832  0.000000E+00  0.000000E+00      -17.7766       85.2537       3.90356        1       Scatag_D
       4       1946.02       5763.49  0.000000E+00  0.000000E+00       2601.49  0.000000E+00       2648.79       9301.52  0.000000E+00       13311.0       55.7354       11348.7 -1.000000E+10 -1.000000E+10 -1.000000E+10 -1.000000E+10 -1.000000E+10  0.000000E+00  0.000000E+00  0.000000E+00  0.000000E+00  0.000000E+00  0.000000E+00  0.000000E+00  0.000000E+00  0.000000E+00  0.000000E+00  0.000000E+00  0.000000E+00       Funtag_D
       5  4.763006E+05  4.294361E+05       46864.5       25.0883    0.00226111     0.0288388      0.184725      0.239716  9.798286E-06   -0.00109577    0.00830113       1.02748       Opttag_D
       5       83.3993       1.13849       1.00000  0.000000E+00       1.00000  0.000000E+00      0.947820       89.4022       1.00000       37.7089  0.000000E+00       8.88720  0.000000E+00       8.88720  0.000000E+00  0.000000E+00      -17.7744       85.2537       3.90356        1       Scatag_D
       5       1942.08       5764.22  0.000000E+00  0.000000E+00       2601.40  0.000000E+00       2645.84       9221.90  0.000000E+00       13304.0       55.7393       11329.4 -1.000000E+10 -1.000000E+10 -1.000000E+10 -1.000000E+10 -1.000000E+10  0.000000E+00  0.000000E+00  0.000000E+00  0.000000E+00  0.000000E+00  0.000000E+00  0.000000E+00  0.000000E+00  0.000000E+00  0.000000E+00  0.000000E+00  0.000000E+00       Funtag_D
       6  4.762421E+05  4.294430E+05       46799.1       30.2055    0.00303818     0.0390753      0.184723      0.239756 -7.877054E-06   -0.00128536    0.00829731       1.02864       Opttag_D
       6       83.4016       1.13892       1.00000  0.000000E+00       1.00000  0.000000E+00      0.947835       89.3842       1.00000       37.6687  0.000000E+00       8.88597  0.000000E+00       8.88597  0.000000E+00  0.000000E+00      -17.7719       85.2537       3.90356        1       Scatag_D
       6       1940.29       5777.19  0.000000E+00  0.000000E+00       2601.20  0.000000E+00       2642.84       9170.43  0.000000E+00       13296.1       55.7491       11315.3 -1.000000E+10 -1.000000E+10 -1.000000E+10 -1.000000E+10 -1.000000E+10  0.000000E+00  0.000000E+00  0.000000E+00  0.000000E+00  0.000000E+00  0.000000E+00  0.000000E+00  0.000000E+00  0.000000E+00  0.000000E+00  0.000000E+00  0.000000E+00       Funtag_D
       7  4.761871E+05  4.294369E+05       46750.3       21.3141    0.00322503     0.0416650      0.184719      0.239764  7.684930E-06   -0.00135126    0.00828965       1.02697       Opttag_D
       7       83.4021       1.13882       1.00000  0.000000E+00       1.00000  0.000000E+00      0.947840       89.3805       1.00000       37.6579  0.000000E+00       8.88563  0.000000E+00       8.88563  0.000000E+00  0.000000E+00      -17.7713       85.2537       3.90356        1       Scatag_D
       7       1936.71       5758.51  0.000000E+00  0.000000E+00       2601.23  0.000000E+00       2641.90       9149.83  0.000000E+00       13294.8       55.7474       11311.6 -1.000000E+10 -1.000000E+10 -1.000000E+10 -1.000000E+10 -1.000000E+10  0.000000E+00  0.000000E+00  0.000000E+00  0.000000E+00  0.000000E+00  0.000000E+00  0.000000E+00  0.000000E+00  0.000000E+00  0.000000E+00  0.000000E+00  0.000000E+00       Funtag_D
       8  4.761458E+05  4.294262E+05       46719.6       20.1304    0.00345655     0.0448306      0.184713      0.239775  3.496643E-05   -0.00143932    0.00829016       1.02681       Opttag_D
       8       83.4026       1.13860       1.00000  0.000000E+00       1.00000  0.000000E+00      0.947846       89.3765       1.00000       37.6448  0.000000E+00       8.88517  0.000000E+00       8.88517  0.000000E+00  0.000000E+00      -17.7703       85.2537       3.90356        1       Scatag_D
       8       1936.95       5756.62  0.000000E+00  0.000000E+00       2601.26  0.000000E+00       2640.69       9127.18  0.000000E+00       13293.1       55.7342       11308.1 -1.000000E+10 -1.000000E+10 -1.000000E+10 -1.000000E+10 -1.000000E+10  0.000000E+00  0.000000E+00  0.000000E+00  0.000000E+00  0.000000E+00  0.000000E+00  0.000000E+00  0.000000E+00  0.000000E+00  0.000000E+00  0.000000E+00  0.000000E+00       Funtag_D
       9  4.760913E+05  4.294108E+05       46680.5       22.0528    0.00405841     0.0528296      0.184700      0.239799  7.425564E-05   -0.00161947    0.00829124       1.02685       Opttag_D
       9       83.4039       1.13833       1.00000  0.000000E+00       1.00000  0.000000E+00      0.947863       89.3640       1.00000       37.6138  0.000000E+00       8.88410  0.000000E+00       8.88410  0.000000E+00  0.000000E+00      -17.7682       85.2537       3.90356        1       Scatag_D
       9       1937.45       5757.14  0.000000E+00  0.000000E+00       2601.23  0.000000E+00       2638.17       9100.80  0.000000E+00       13289.2       55.6960       11300.9 -1.000000E+10 -1.000000E+10 -1.000000E+10 -1.000000E+10 -1.000000E+10  0.000000E+00  0.000000E+00  0.000000E+00  0.000000E+00  0.000000E+00  0.000000E+00  0.000000E+00  0.000000E+00  0.000000E+00  0.000000E+00  0.000000E+00  0.000000E+00       Funtag_D
      10  4.760457E+05  4.293890E+05       46656.8       33.8156    0.00547453     0.0711125      0.184682      0.239850  1.298753E-04   -0.00198821    0.00830372       1.02864       Opttag_D
      10       83.4068       1.13804       1.00000  0.000000E+00       1.00000  0.000000E+00      0.947904       89.3327       1.00000       37.5461  0.000000E+00       8.88144  0.000000E+00       8.88144  0.000000E+00  0.000000E+00      -17.7629       85.2537       3.90356        1       Scatag_D
      10       1943.29       5777.24  0.000000E+00  0.000000E+00       2601.17  0.000000E+00       2633.57       9076.60  0.000000E+00       13281.3       55.6087       11288.0 -1.000000E+10 -1.000000E+10 -1.000000E+10 -1.000000E+10 -1.000000E+10  0.000000E+00  0.000000E+00  0.000000E+00  0.000000E+00  0.000000E+00  0.000000E+00  0.000000E+00  0.000000E+00  0.000000E+00  0.000000E+00  0.000000E+00  0.000000E+00       Funtag_D
      11  4.759775E+05  4.293841E+05       46593.5       20.6613    0.00590761     0.0766597      0.184680      0.239863  1.424594E-04   -0.00209291    0.00829193       1.02598       Opttag_D
      11       83.4077       1.13795       1.00000  0.000000E+00       1.00000  0.000000E+00      0.947918       89.3203       1.00000       37.5258  0.000000E+00       8.88064  0.000000E+00       8.88064  0.000000E+00  0.000000E+00      -17.7613       85.2537       3.90356        1       Scatag_D
      11       1937.78       5747.40  0.000000E+00  0.000000E+00       2601.07  0.000000E+00       2632.68       9056.24  0.000000E+00       13279.2       55.5702       11283.5 -1.000000E+10 -1.000000E+10 -1.000000E+10 -1.000000E+10 -1.000000E+10  0.000000E+00  0.000000E+00  0.000000E+00  0.000000E+00  0.000000E+00  0.000000E+00  0.000000E+00  0.000000E+00  0.000000E+00  0.000000E+00  0.000000E+00  0.000000E+00       Funtag_D
      12  4.759275E+05  4.293788E+05       46548.7       18.0381    0.00653990     0.0845920      0.184673      0.239881  1.558120E-04   -0.00223066    0.00828582       1.02597       Opttag_D
      12       83.4090       1.13789       1.00000  0.000000E+00       1.00000  0.000000E+00      0.947938       89.3021       1.00000       37.4962  0.000000E+00       8.87938  0.000000E+00       8.87938  0.000000E+00  0.000000E+00      -17.7588       85.2537       3.90356        1       Scatag_D
      12       1934.92       5747.31  0.000000E+00  0.000000E+00       2600.91  0.000000E+00       2631.76       9025.97  0.000000E+00       13276.4       55.5176       11276.0 -1.000000E+10 -1.000000E+10 -1.000000E+10 -1.000000E+10 -1.000000E+10  0.000000E+00  0.000000E+00  0.000000E+00  0.000000E+00  0.000000E+00  0.000000E+00  0.000000E+00  0.000000E+00  0.000000E+00  0.000000E+00  0.000000E+00  0.000000E+00       Funtag_D
      13  4.758833E+05  4.293718E+05       46511.5       18.2505    0.00744782     0.0959139      0.184666      0.239908  1.736794E-04   -0.00242195    0.00828189       1.02598       Opttag_D
      13       83.4108       1.13784       1.00000  0.000000E+00       1.00000  0.000000E+00      0.947967       89.2770       1.00000       37.4545  0.000000E+00       8.87758  0.000000E+00       8.87758  0.000000E+00  0.000000E+00      -17.7552       85.2537       3.90356        1       Scatag_D
      13       1933.08       5747.34  0.000000E+00  0.000000E+00       2600.73  0.000000E+00       2630.90       9006.20  0.000000E+00       13272.9       55.4682       11264.9 -1.000000E+10 -1.000000E+10 -1.000000E+10 -1.000000E+10 -1.000000E+10  0.000000E+00  0.000000E+00  0.000000E+00  0.000000E+00  0.000000E+00  0.000000E+00  0.000000E+00  0.000000E+00  0.000000E+00  0.000000E+00  0.000000E+00  0.000000E+00       Funtag_D
      14  4.758747E+05  4.293537E+05       46521.0       42.1068    0.00961303      0.125307      0.184650      0.239962  2.197890E-04   -0.00283646    0.00829580       1.03119       Opttag_D
      14       83.4147       1.13771       1.00000  0.000000E+00       1.00000  0.000000E+00      0.948034       89.2202       1.00000       37.3608  0.000000E+00       8.87356  0.000000E+00       8.87356  0.000000E+00  0.000000E+00      -17.7471       85.2537       3.90356        1       Scatag_D
      14       1939.58       5805.90  0.000000E+00  0.000000E+00       2600.38  0.000000E+00       2630.45       8985.63  0.000000E+00       13265.6       55.4137       11238.1 -1.000000E+10 -1.000000E+10 -1.000000E+10 -1.000000E+10 -1.000000E+10  0.000000E+00  0.000000E+00  0.000000E+00  0.000000E+00  0.000000E+00  0.000000E+00  0.000000E+00  0.000000E+00  0.000000E+00  0.000000E+00  0.000000E+00  0.000000E+00       Funtag_D
      15  4.757945E+05  4.293523E+05       46442.1       18.3250    0.00971188      0.127717      0.184649      0.239962  2.233433E-04   -0.00285445    0.00827973       1.02551       Opttag_D
      15       83.4149       1.13771       1.00000  0.000000E+00       1.00000  0.000000E+00      0.948036       89.2186       1.00000       37.3561  0.000000E+00       8.87339  0.000000E+00       8.87339  0.000000E+00  0.000000E+00      -17.7468       85.2537       3.90356        1       Scatag_D
      15       1932.07       5742.06  0.000000E+00  0.000000E+00       2600.35  0.000000E+00       2630.67       8981.42  0.000000E+00       13265.4       55.4119       11234.7 -1.000000E+10 -1.000000E+10 -1.000000E+10 -1.000000E+10 -1.000000E+10  0.000000E+00  0.000000E+00  0.000000E+00  0.000000E+00  0.000000E+00  0.000000E+00  0.000000E+00  0.000000E+00  0.000000E+00  0.000000E+00  0.000000E+00  0.000000E+00       Funtag_D
      16  4.757659E+05  4.293463E+05       46419.7       14.9096     0.0100031      0.134158      0.184644      0.239964  2.389053E-04   -0.00289798    0.00827998       1.02510       Opttag_D
      16       83.4152       1.13765       1.00000  0.000000E+00       1.00000  0.000000E+00      0.948043       89.2139       1.00000       37.3434  0.000000E+00       8.87297  0.000000E+00       8.87297  0.000000E+00  0.000000E+00      -17.7459       85.2537       3.90356        1       Scatag_D
      16       1932.19       5737.45  0.000000E+00  0.000000E+00       2600.31  0.000000E+00       2630.98       8970.43  0.000000E+00       13264.6       55.4126       11228.3 -1.000000E+10 -1.000000E+10 -1.000000E+10 -1.000000E+10 -1.000000E+10  0.000000E+00  0.000000E+00  0.000000E+00  0.000000E+00  0.000000E+00  0.000000E+00  0.000000E+00  0.000000E+00  0.000000E+00  0.000000E+00  0.000000E+00  0.000000E+00       Funtag_D
      17  4.757281E+05  4.293327E+05       46395.4       16.9104     0.0109057      0.152907      0.184634      0.239979  2.733914E-04   -0.00303776    0.00828383       1.02521       Opttag_D
      17       83.4165       1.13751       1.00000  0.000000E+00       1.00000  0.000000E+00      0.948066       89.1960       1.00000       37.3066  0.000000E+00       8.87164  0.000000E+00       8.87164  0.000000E+00  0.000000E+00      -17.7433       85.2537       3.90356        1       Scatag_D
      17       1933.99       5738.72  0.000000E+00  0.000000E+00       2600.17  0.000000E+00       2631.71       8962.14  0.000000E+00       13262.2       55.4031       11211.0 -1.000000E+10 -1.000000E+10 -1.000000E+10 -1.000000E+10 -1.000000E+10  0.000000E+00  0.000000E+00  0.000000E+00  0.000000E+00  0.000000E+00  0.000000E+00  0.000000E+00  0.000000E+00  0.000000E+00  0.000000E+00  0.000000E+00  0.000000E+00       Funtag_D
      18  4.756745E+05  4.293131E+05       46361.4       18.8870     0.0122435      0.183943      0.184619      0.239998  3.234395E-04   -0.00322803    0.00828956       1.02540       Opttag_D
      18       83.4183       1.13727       1.00000  0.000000E+00       1.00000  0.000000E+00      0.948097       89.1704       1.00000       37.2561  0.000000E+00       8.86980  0.000000E+00       8.86980  0.000000E+00  0.000000E+00      -17.7396       85.2537       3.90356        1       Scatag_D
      18       1936.67       5740.90  0.000000E+00  0.000000E+00       2599.98  0.000000E+00       2632.89       8955.71  0.000000E+00       13259.2       55.3643       11180.7 -1.000000E+10 -1.000000E+10 -1.000000E+10 -1.000000E+10 -1.000000E+10  0.000000E+00  0.000000E+00  0.000000E+00  0.000000E+00  0.000000E+00  0.000000E+00  0.000000E+00  0.000000E+00  0.000000E+00  0.000000E+00  0.000000E+00  0.000000E+00       Funtag_D
      19  4.756501E+05  4.292813E+05       46368.9       40.4146     0.0161357      0.275937      0.184596      0.240049  4.046116E-04   -0.00375747    0.00831649       1.02974       Opttag_D
      19       83.4237       1.13695       1.00000  0.000000E+00       1.00000  0.000000E+00      0.948186       89.0884       1.00000       37.1269  0.000000E+00       8.86443  0.000000E+00       8.86443  0.000000E+00  0.000000E+00      -17.7289       85.2537       3.90356        1       Scatag_D
      19       1949.27       5789.62  0.000000E+00  0.000000E+00       2599.50  0.000000E+00       2637.77       9006.19  0.000000E+00       13255.0       55.2731       11076.3 -1.000000E+10 -1.000000E+10 -1.000000E+10 -1.000000E+10 -1.000000E+10  0.000000E+00  0.000000E+00  0.000000E+00  0.000000E+00  0.000000E+00  0.000000E+00  0.000000E+00  0.000000E+00  0.000000E+00  0.000000E+00  0.000000E+00  0.000000E+00       Funtag_D
      20  4.755510E+05  4.292764E+05       46274.6       19.7642     0.0163290      0.286159      0.184593      0.240051  4.171956E-04   -0.00377187    0.00830053       1.02503       Opttag_D
      20       83.4237       1.13685       1.00000  0.000000E+00       1.00000  0.000000E+00      0.948186       89.0880       1.00000       37.1226  0.000000E+00       8.86424  0.000000E+00       8.86424  0.000000E+00  0.000000E+00      -17.7285       85.2537       3.90356        1       Scatag_D
      20       1941.79       5736.73  0.000000E+00  0.000000E+00       2599.53  0.000000E+00       2637.84       8987.86  0.000000E+00       13254.9       55.2613       11060.7 -1.000000E+10 -1.000000E+10 -1.000000E+10 -1.000000E+10 -1.000000E+10  0.000000E+00  0.000000E+00  0.000000E+00  0.000000E+00  0.000000E+00  0.000000E+00  0.000000E+00  0.000000E+00  0.000000E+00  0.000000E+00  0.000000E+00  0.000000E+00       Funtag_D
      21  4.754945E+05  4.292698E+05       46224.7       17.9131     0.0167896      0.305992      0.184589      0.240057  4.338143E-04   -0.00381437    0.00829723       1.02505       Opttag_D
      21       83.4237       1.13669       1.00000  0.000000E+00       1.00000  0.000000E+00      0.948191       89.0816       1.00000       37.1118  0.000000E+00       8.86363  0.000000E+00       8.86363  0.000000E+00  0.000000E+00      -17.7273       85.2537       3.90356        1       Scatag_D
      21       1940.25       5736.99  0.000000E+00  0.000000E+00       2599.46  0.000000E+00       2638.07       8967.98  0.000000E+00       13254.7       55.2465       11032.0 -1.000000E+10 -1.000000E+10 -1.000000E+10 -1.000000E+10 -1.000000E+10  0.000000E+00  0.000000E+00  0.000000E+00  0.000000E+00  0.000000E+00  0.000000E+00  0.000000E+00  0.000000E+00  0.000000E+00  0.000000E+00  0.000000E+00  0.000000E+00       Funtag_D
      22  4.754072E+05  4.292561E+05       46151.2       24.5949     0.0188749      0.377991      0.184583      0.240081  4.689729E-04   -0.00403389    0.00829894       1.02617       Opttag_D
      22       83.4251       1.13643       1.00000  0.000000E+00       1.00000  0.000000E+00      0.948221       89.0456       1.00000       37.0606  0.000000E+00       8.86084  0.000000E+00       8.86084  0.000000E+00  0.000000E+00      -17.7217       85.2537       3.90356        1       Scatag_D
      22       1941.05       5749.46  0.000000E+00  0.000000E+00       2599.28  0.000000E+00       2640.38       8973.33  0.000000E+00       13255.1       55.2420       10937.3 -1.000000E+10 -1.000000E+10 -1.000000E+10 -1.000000E+10 -1.000000E+10  0.000000E+00  0.000000E+00  0.000000E+00  0.000000E+00  0.000000E+00  0.000000E+00  0.000000E+00  0.000000E+00  0.000000E+00  0.000000E+00  0.000000E+00  0.000000E+00       Funtag_D
      23  4.753629E+05  4.292399E+05       46123.1       38.9819     0.0202662      0.439308      0.184573      0.240091  5.102794E-04   -0.00410912    0.00831668       1.03001       Opttag_D
      23       83.4238       1.13597       1.00000  0.000000E+00       1.00000  0.000000E+00      0.948224       89.0302       1.00000       37.0371  0.000000E+00       8.85929  0.000000E+00       8.85929  0.000000E+00  0.000000E+00      -17.7186       85.2537       3.90356        1       Scatag_D
      23       1949.36       5792.61  0.000000E+00  0.000000E+00       2598.95  0.000000E+00       2639.41       8957.70  0.000000E+00       13255.9       55.3312       10873.8 -1.000000E+10 -1.000000E+10 -1.000000E+10 -1.000000E+10 -1.000000E+10  0.000000E+00  0.000000E+00  0.000000E+00  0.000000E+00  0.000000E+00  0.000000E+00  0.000000E+00  0.000000E+00  0.000000E+00  0.000000E+00  0.000000E+00  0.000000E+00       Funtag_D
      24  4.752733E+05  4.292383E+05       46035.1       21.1914     0.0202443      0.441525      0.184573      0.240087  5.143139E-04   -0.00407628    0.00829312       1.02536       Opttag_D
      24       83.4229       1.13592       1.00000  0.000000E+00       1.00000  0.000000E+00      0.948217       89.0336       1.00000       37.0400  0.000000E+00       8.85931  0.000000E+00       8.85931  0.000000E+00  0.000000E+00      -17.7186       85.2537       3.90356        1       Scatag_D
      24       1938.33       5740.43  0.000000E+00  0.000000E+00       2599.03  0.000000E+00       2638.05       8944.59  0.000000E+00       13255.3       55.3749       10864.0 -1.000000E+10 -1.000000E+10 -1.000000E+10 -1.000000E+10 -1.000000E+10  0.000000E+00  0.000000E+00  0.000000E+00  0.000000E+00  0.000000E+00  0.000000E+00  0.000000E+00  0.000000E+00  0.000000E+00  0.000000E+00  0.000000E+00  0.000000E+00       Funtag_D
      25  4.752231E+05  4.292344E+05       45988.7       15.3461     0.0200313      0.439648      0.184572      0.240078  5.242083E-04   -0.00399644    0.00828804       1.02443       Opttag_D
      25       83.4210       1.13577       1.00000  0.000000E+00       1.00000  0.000000E+00      0.948200       89.0437       1.00000       37.0501  0.000000E+00       8.85978  0.000000E+00       8.85978  0.000000E+00  0.000000E+00      -17.7196       85.2537       3.90356        1       Scatag_D
      25       1935.95       5730.04  0.000000E+00  0.000000E+00       2599.10  0.000000E+00       2635.39       8928.91  0.000000E+00       13254.8       55.4366       10849.1 -1.000000E+10 -1.000000E+10 -1.000000E+10 -1.000000E+10 -1.000000E+10  0.000000E+00  0.000000E+00  0.000000E+00  0.000000E+00  0.000000E+00  0.000000E+00  0.000000E+00  0.000000E+00  0.000000E+00  0.000000E+00  0.000000E+00  0.000000E+00       Funtag_D
      26  4.751779E+05  4.292265E+05       45951.4       16.8430     0.0205812      0.459898      0.184565      0.240076  5.442852E-04   -0.00398564    0.00828483       1.02449       Opttag_D
      26       83.4193       1.13555       1.00000  0.000000E+00       1.00000  0.000000E+00      0.948190       89.0441       1.00000       37.0443  0.000000E+00       8.85951  0.000000E+00       8.85951  0.000000E+00  0.000000E+00      -17.7190       85.2537       3.90356        1       Scatag_D
      26       1934.46       5730.65  0.000000E+00  0.000000E+00       2599.10  0.000000E+00       2633.29       8926.27  0.000000E+00       13255.0       55.4915       10817.1 -1.000000E+10 -1.000000E+10 -1.000000E+10 -1.000000E+10 -1.000000E+10  0.000000E+00  0.000000E+00  0.000000E+00  0.000000E+00  0.000000E+00  0.000000E+00  0.000000E+00  0.000000E+00  0.000000E+00  0.000000E+00  0.000000E+00  0.000000E+00       Funtag_D
      27  4.751299E+05  4.292124E+05       45917.5       26.6667     0.0214142      0.475245      0.184556      0.240062  5.805004E-04   -0.00392705    0.00829438       1.02589       Opttag_D
      27       83.4146       1.13512       1.00000  0.000000E+00       1.00000  0.000000E+00      0.948158       89.0531       1.00000       37.0401  0.000000E+00       8.85961  0.000000E+00       8.85961  0.000000E+00  0.000000E+00      -17.7192       85.2537       3.90356        1       Scatag_D
      27       1938.92       5746.36  0.000000E+00  0.000000E+00       2599.21  0.000000E+00       2630.96       8916.53  0.000000E+00       13255.6       55.5329       10774.4 -1.000000E+10 -1.000000E+10 -1.000000E+10 -1.000000E+10 -1.000000E+10  0.000000E+00  0.000000E+00  0.000000E+00  0.000000E+00  0.000000E+00  0.000000E+00  0.000000E+00  0.000000E+00  0.000000E+00  0.000000E+00  0.000000E+00  0.000000E+00       Funtag_D
      28  4.750881E+05  4.292098E+05       45878.3       17.9430     0.0212823      0.465963      0.184553      0.240064  5.869365E-04   -0.00394640    0.00828362       1.02450       Opttag_D
      28       83.4138       1.13502       1.00000  0.000000E+00       1.00000  0.000000E+00      0.948152       89.0562       1.00000       37.0411  0.000000E+00       8.86007  0.000000E+00       8.86007  0.000000E+00  0.000000E+00      -17.7201       85.2537       3.90356        1       Scatag_D
      28       1933.89       5730.81  0.000000E+00  0.000000E+00       2599.12  0.000000E+00       2631.19       8906.53  0.000000E+00       13254.7       55.5402       10766.6 -1.000000E+10 -1.000000E+10 -1.000000E+10 -1.000000E+10 -1.000000E+10  0.000000E+00  0.000000E+00  0.000000E+00  0.000000E+00  0.000000E+00  0.000000E+00  0.000000E+00  0.000000E+00  0.000000E+00  0.000000E+00  0.000000E+00  0.000000E+00       Funtag_D
      29  4.750665E+05  4.292116E+05       45854.9       9.88565     0.0210331      0.449678      0.184554      0.240065  5.823256E-04   -0.00396315    0.00828104       1.02359       Opttag_D
      29       83.4145       1.13508       1.00000  0.000000E+00       1.00000  0.000000E+00      0.948158       89.0549       1.00000       37.0420  0.000000E+00       8.86018  0.000000E+00       8.86018  0.000000E+00  0.000000E+00      -17.7204       85.2537       3.90356        1       Scatag_D
      29       1932.69       5720.59  0.000000E+00  0.000000E+00       2599.15  0.000000E+00       2631.13       8896.52  0.000000E+00       13254.2       55.4724       10765.2 -1.000000E+10 -1.000000E+10 -1.000000E+10 -1.000000E+10 -1.000000E+10  0.000000E+00  0.000000E+00  0.000000E+00  0.000000E+00  0.000000E+00  0.000000E+00  0.000000E+00  0.000000E+00  0.000000E+00  0.000000E+00  0.000000E+00  0.000000E+00       Funtag_D
      30  4.750546E+05  4.292085E+05       45846.2       8.89000     0.0211646      0.458468      0.184551      0.240064  5.903948E-04   -0.00398058    0.00828106       1.02349       Opttag_D
      30       83.4138       1.13497       1.00000  0.000000E+00       1.00000  0.000000E+00      0.948156       89.0544       1.00000       37.0395  0.000000E+00       8.86016  0.000000E+00       8.86016  0.000000E+00  0.000000E+00      -17.7203       85.2537       3.90356        1       Scatag_D
      30       1932.69       5719.46  0.000000E+00  0.000000E+00       2599.14  0.000000E+00       2631.25       8892.98  0.000000E+00       13253.7       55.4534       10761.4 -1.000000E+10 -1.000000E+10 -1.000000E+10 -1.000000E+10 -1.000000E+10  0.000000E+00  0.000000E+00  0.000000E+00  0.000000E+00  0.000000E+00  0.000000E+00  0.000000E+00  0.000000E+00  0.000000E+00  0.000000E+00  0.000000E+00  0.000000E+00       Funtag_D
      31  4.750338E+05  4.291998E+05       45834.0       9.03977     0.0214499      0.474659      0.184546      0.240062  6.124889E-04   -0.00403872    0.00828255       1.02350       Opttag_D
      31       83.4121       1.13469       1.00000  0.000000E+00       1.00000  0.000000E+00      0.948153       89.0523       1.00000       37.0337  0.000000E+00       8.86011  0.000000E+00       8.86011  0.000000E+00  0.000000E+00      -17.7202       85.2537       3.90356        1       Scatag_D
      31       1933.39       5719.61  0.000000E+00  0.000000E+00       2599.18  0.000000E+00       2631.44       8886.42  0.000000E+00       13252.4       55.4120       10756.2 -1.000000E+10 -1.000000E+10 -1.000000E+10 -1.000000E+10 -1.000000E+10  0.000000E+00  0.000000E+00  0.000000E+00  0.000000E+00  0.000000E+00  0.000000E+00  0.000000E+00  0.000000E+00  0.000000E+00  0.000000E+00  0.000000E+00  0.000000E+00       Funtag_D
      32  4.750235E+05  4.291846E+05       45838.9       15.2765     0.0218686      0.490677      0.184536      0.240055  6.512018E-04   -0.00411654    0.00828666       1.02413       Opttag_D
      32       83.4090       1.13419       1.00000  0.000000E+00       1.00000  0.000000E+00      0.948148       89.0500       1.00000       37.0268  0.000000E+00       8.86029  0.000000E+00       8.86029  0.000000E+00  0.000000E+00      -17.7206       85.2537       3.90356        1       Scatag_D
      32       1935.31       5726.68  0.000000E+00  0.000000E+00       2599.22  0.000000E+00       2631.29       8883.61  0.000000E+00       13251.2       55.3728       10756.2 -1.000000E+10 -1.000000E+10 -1.000000E+10 -1.000000E+10 -1.000000E+10  0.000000E+00  0.000000E+00  0.000000E+00  0.000000E+00  0.000000E+00  0.000000E+00  0.000000E+00  0.000000E+00  0.000000E+00  0.000000E+00  0.000000E+00  0.000000E+00       Funtag_D
      33  4.750063E+05  4.291791E+05       45827.2       7.60083     0.0220001      0.492844      0.184533      0.240052  6.653228E-04   -0.00414601    0.00828155       1.02337       Opttag_D
      33       83.4080       1.13401       1.00000  0.000000E+00       1.00000  0.000000E+00      0.948147       89.0488       1.00000       37.0244  0.000000E+00       8.86036  0.000000E+00       8.86036  0.000000E+00  0.000000E+00      -17.7207       85.2537       3.90356        1       Scatag_D
      33       1932.92       5718.17  0.000000E+00  0.000000E+00       2599.27  0.000000E+00       2631.22       8883.17  0.000000E+00       13250.7       55.3740       10756.4 -1.000000E+10 -1.000000E+10 -1.000000E+10 -1.000000E+10 -1.000000E+10  0.000000E+00  0.000000E+00  0.000000E+00  0.000000E+00  0.000000E+00  0.000000E+00  0.000000E+00  0.000000E+00  0.000000E+00  0.000000E+00  0.000000E+00  0.000000E+00       Funtag_D
      34  4.749972E+05  4.291735E+05       45823.7       6.71469     0.0220702      0.488670      0.184530      0.240049  6.796360E-04   -0.00416928    0.00828006       1.02331       Opttag_D
      34       83.4069       1.13383       1.00000  0.000000E+00       1.00000  0.000000E+00      0.948147       89.0481       1.00000       37.0230  0.000000E+00       8.86052  0.000000E+00       8.86052  0.000000E+00  0.000000E+00      -17.7210       85.2537       3.90356        1       Scatag_D
      34       1932.23       5717.52  0.000000E+00  0.000000E+00       2599.31  0.000000E+00       2631.04       8882.97  0.000000E+00       13250.5       55.3845       10754.7 -1.000000E+10 -1.000000E+10 -1.000000E+10 -1.000000E+10 -1.000000E+10  0.000000E+00  0.000000E+00  0.000000E+00  0.000000E+00  0.000000E+00  0.000000E+00  0.000000E+00  0.000000E+00  0.000000E+00  0.000000E+00  0.000000E+00  0.000000E+00       Funtag_D
      35  4.749864E+05  4.291628E+05       45823.6       8.15990     0.0222279      0.482196      0.184525      0.240043  7.068214E-04   -0.00421314    0.00827982       1.02336       Opttag_D
      35       83.4048       1.13347       1.00000  0.000000E+00       1.00000  0.000000E+00      0.948147       89.0466       1.00000       37.0197  0.000000E+00       8.86086  0.000000E+00       8.86086  0.000000E+00  0.000000E+00      -17.7217       85.2537       3.90356        1       Scatag_D
      35       1932.12       5718.06  0.000000E+00  0.000000E+00       2599.37  0.000000E+00       2630.92       8884.23  0.000000E+00       13250.3       55.4033       10753.2 -1.000000E+10 -1.000000E+10 -1.000000E+10 -1.000000E+10 -1.000000E+10  0.000000E+00  0.000000E+00  0.000000E+00  0.000000E+00  0.000000E+00  0.000000E+00  0.000000E+00  0.000000E+00  0.000000E+00  0.000000E+00  0.000000E+00  0.000000E+00       Funtag_D
      36  4.749691E+05  4.291415E+05       45827.6       10.5303     0.0226024      0.469807      0.184515      0.240031  7.611924E-04   -0.00431109    0.00828175       1.02346       Opttag_D
      36       83.4000       1.13272       1.00000  0.000000E+00       1.00000  0.000000E+00      0.948145       89.0426       1.00000       37.0115  0.000000E+00       8.86158  0.000000E+00       8.86158  0.000000E+00  0.000000E+00      -17.7232       85.2537       3.90356        1       Scatag_D
      36       1933.02       5719.11  0.000000E+00  0.000000E+00       2599.46  0.000000E+00       2631.01       8885.94  0.000000E+00       13250.2       55.4389       10753.4 -1.000000E+10 -1.000000E+10 -1.000000E+10 -1.000000E+10 -1.000000E+10  0.000000E+00  0.000000E+00  0.000000E+00  0.000000E+00  0.000000E+00  0.000000E+00  0.000000E+00  0.000000E+00  0.000000E+00  0.000000E+00  0.000000E+00  0.000000E+00       Funtag_D
      37  4.749547E+05  4.291126E+05       45842.1       10.3731     0.0232357      0.467256      0.184500      0.240017  8.348716E-04   -0.00445537    0.00828206       1.02344       Opttag_D
      37       83.3930       1.13168       1.00000  0.000000E+00       1.00000  0.000000E+00      0.948145       89.0353       1.00000       36.9991  0.000000E+00       8.86280  0.000000E+00       8.86280  0.000000E+00  0.000000E+00      -17.7256       85.2537       3.90356        1       Scatag_D
      37       1933.16       5718.94  0.000000E+00  0.000000E+00       2599.49  0.000000E+00       2631.34       8892.52  0.000000E+00       13250.8       55.5301       10760.3 -1.000000E+10 -1.000000E+10 -1.000000E+10 -1.000000E+10 -1.000000E+10  0.000000E+00  0.000000E+00  0.000000E+00  0.000000E+00  0.000000E+00  0.000000E+00  0.000000E+00  0.000000E+00  0.000000E+00  0.000000E+00  0.000000E+00  0.000000E+00       Funtag_D
      38  4.749416E+05  4.291096E+05       45832.0       6.40713     0.0233406      0.467550      0.184498      0.240016  8.425565E-04   -0.00447381    0.00828141       1.02319       Opttag_D
      38       83.3918       1.13154       1.00000  0.000000E+00       1.00000  0.000000E+00      0.948145       89.0333       1.00000       36.9960  0.000000E+00       8.86298  0.000000E+00       8.86298  0.000000E+00  0.000000E+00      -17.7260       85.2537       3.90356        1       Scatag_D
      38       1932.86       5716.14  0.000000E+00  0.000000E+00       2599.52  0.000000E+00       2630.81       8887.62  0.000000E+00       13250.8       55.5132       10758.8 -1.000000E+10 -1.000000E+10 -1.000000E+10 -1.000000E+10 -1.000000E+10  0.000000E+00  0.000000E+00  0.000000E+00  0.000000E+00  0.000000E+00  0.000000E+00  0.000000E+00  0.000000E+00  0.000000E+00  0.000000E+00  0.000000E+00  0.000000E+00       Funtag_D
      39  4.749291E+05  4.291034E+05       45825.7       6.96542     0.0235796      0.469217      0.184493      0.240015  8.585028E-04   -0.00451430    0.00828219       1.02317       Opttag_D
      39       83.3893       1.13127       1.00000  0.000000E+00       1.00000  0.000000E+00      0.948146       89.0289       1.00000       36.9892  0.000000E+00       8.86341  0.000000E+00       8.86341  0.000000E+00  0.000000E+00      -17.7268       85.2537       3.90356        1       Scatag_D
      39       1933.23       5715.96  0.000000E+00  0.000000E+00       2599.52  0.000000E+00       2629.92       8884.72  0.000000E+00       13250.8       55.4996       10756.1 -1.000000E+10 -1.000000E+10 -1.000000E+10 -1.000000E+10 -1.000000E+10  0.000000E+00  0.000000E+00  0.000000E+00  0.000000E+00  0.000000E+00  0.000000E+00  0.000000E+00  0.000000E+00  0.000000E+00  0.000000E+00  0.000000E+00  0.000000E+00       Funtag_D
      40  4.749172E+05  4.290920E+05       45825.2       7.94632     0.0240232      0.472592      0.184486      0.240011  8.875134E-04   -0.00458402    0.00828229       1.02320       Opttag_D
      40       83.3852       1.13078       1.00000  0.000000E+00       1.00000  0.000000E+00      0.948148       89.0215       1.00000       36.9790  0.000000E+00       8.86414  0.000000E+00       8.86414  0.000000E+00  0.000000E+00      -17.7283       85.2537       3.90356        1       Scatag_D
      40       1933.27       5716.25  0.000000E+00  0.000000E+00       2599.54  0.000000E+00       2629.30       8885.34  0.000000E+00       13251.0       55.5278       10755.0 -1.000000E+10 -1.000000E+10 -1.000000E+10 -1.000000E+10 -1.000000E+10  0.000000E+00  0.000000E+00  0.000000E+00  0.000000E+00  0.000000E+00  0.000000E+00  0.000000E+00  0.000000E+00  0.000000E+00  0.000000E+00  0.000000E+00  0.000000E+00       Funtag_D
      41  4.749027E+05  4.290641E+05       45838.6       12.3472     0.0252934      0.482698      0.184469      0.240005  9.585990E-04   -0.00476035    0.00828616       1.02348       Opttag_D
      41       83.3742       1.12956       1.00000  0.000000E+00       1.00000  0.000000E+00      0.948152       89.0020       1.00000       36.9535  0.000000E+00       8.86607  0.000000E+00       8.86607  0.000000E+00  0.000000E+00      -17.7321       85.2537       3.90356        1       Scatag_D
      41       1935.08       5719.34  0.000000E+00  0.000000E+00       2599.46  0.000000E+00       2629.10       8891.43  0.000000E+00       13252.4       55.6417       10756.1 -1.000000E+10 -1.000000E+10 -1.000000E+10 -1.000000E+10 -1.000000E+10  0.000000E+00  0.000000E+00  0.000000E+00  0.000000E+00  0.000000E+00  0.000000E+00  0.000000E+00  0.000000E+00  0.000000E+00  0.000000E+00  0.000000E+00  0.000000E+00       Funtag_D
      42  4.748863E+05  4.290554E+05       45830.9       7.79329     0.0257749      0.487106      0.184465      0.240004  9.808852E-04   -0.00481242    0.00828271       1.02329       Opttag_D
      42       83.3705       1.12914       1.00000  0.000000E+00       1.00000  0.000000E+00      0.948155       88.9940       1.00000       36.9452  0.000000E+00       8.86671  0.000000E+00       8.86671  0.000000E+00  0.000000E+00      -17.7334       85.2537       3.90356        1       Scatag_D
      42       1933.46       5717.25  0.000000E+00  0.000000E+00       2599.47  0.000000E+00       2628.92       8888.37  0.000000E+00       13253.0       55.7030       10754.7 -1.000000E+10 -1.000000E+10 -1.000000E+10 -1.000000E+10 -1.000000E+10  0.000000E+00  0.000000E+00  0.000000E+00  0.000000E+00  0.000000E+00  0.000000E+00  0.000000E+00  0.000000E+00  0.000000E+00  0.000000E+00  0.000000E+00  0.000000E+00       Funtag_D
      43  4.748743E+05  4.290477E+05       45826.6       7.47686     0.0262222      0.491470      0.184463      0.240004    0.00100048   -0.00485369    0.00828065       1.02333       Opttag_D
      43       83.3671       1.12878       1.00000  0.000000E+00       1.00000  0.000000E+00      0.948157       88.9868       1.00000       36.9378  0.000000E+00       8.86721  0.000000E+00       8.86721  0.000000E+00  0.000000E+00      -17.7344       85.2537       3.90356        1       Scatag_D
      43       1932.51       5717.70  0.000000E+00  0.000000E+00       2599.45  0.000000E+00       2628.46       8887.92  0.000000E+00       13253.5       55.7710       10751.3 -1.000000E+10 -1.000000E+10 -1.000000E+10 -1.000000E+10 -1.000000E+10  0.000000E+00  0.000000E+00  0.000000E+00  0.000000E+00  0.000000E+00  0.000000E+00  0.000000E+00  0.000000E+00  0.000000E+00  0.000000E+00  0.000000E+00  0.000000E+00       Funtag_D
      44  4.748592E+05  4.290328E+05       45826.4       8.52394     0.0271593      0.499346      0.184456      0.240003    0.00103862   -0.00493960    0.00827912       1.02339       Opttag_D
      44       83.3601       1.12804       1.00000  0.000000E+00       1.00000  0.000000E+00      0.948161       88.9722       1.00000       36.9220  0.000000E+00       8.86822  0.000000E+00       8.86822  0.000000E+00  0.000000E+00      -17.7364       85.2537       3.90356        1       Scatag_D
      44       1931.79       5718.39  0.000000E+00  0.000000E+00       2599.37  0.000000E+00       2627.78       8891.48  0.000000E+00       13254.9       55.8857       10746.8 -1.000000E+10 -1.000000E+10 -1.000000E+10 -1.000000E+10 -1.000000E+10  0.000000E+00  0.000000E+00  0.000000E+00  0.000000E+00  0.000000E+00  0.000000E+00  0.000000E+00  0.000000E+00  0.000000E+00  0.000000E+00  0.000000E+00  0.000000E+00       Funtag_D
      45  4.748461E+05  4.290153E+05       45830.8       11.7414     0.0284273      0.508814      0.184450      0.240004    0.00108309   -0.00505993    0.00827896       1.02365       Opttag_D
      45       83.3509       1.12712       1.00000  0.000000E+00       1.00000  0.000000E+00      0.948165       88.9527       1.00000       36.9005  0.000000E+00       8.86953  0.000000E+00       8.86953  0.000000E+00  0.000000E+00      -17.7391       85.2537       3.90356        2       Scatag_D
      45       1931.72       5721.25  0.000000E+00  0.000000E+00       2599.18  0.000000E+00       2627.08       8895.83  0.000000E+00       13256.9       56.0102       10742.8 -1.000000E+10 -1.000000E+10 -1.000000E+10 -1.000000E+10 -1.000000E+10  0.000000E+00  0.000000E+00  0.000000E+00  0.000000E+00  0.000000E+00  0.000000E+00  0.000000E+00  0.000000E+00  0.000000E+00  0.000000E+00  0.000000E+00  0.000000E+00       Funtag_D
      46  4.748286E+05  4.290002E+05       45828.4       8.36506     0.0296309      0.517027      0.184443      0.240004    0.00112162   -0.00516283    0.00827794       1.02355       Opttag_D
      46       83.3426       1.12629       1.00000  0.000000E+00       1.00000  0.000000E+00      0.948169       88.9345       1.00000       36.8807  0.000000E+00       8.87072  0.000000E+00       8.87072  0.000000E+00  0.000000E+00      -17.7414       85.2537       3.90356        1       Scatag_D
      46       1931.24       5720.16  0.000000E+00  0.000000E+00       2598.99  0.000000E+00       2626.69       8895.49  0.000000E+00       13258.9       56.1185       10740.8 -1.000000E+10 -1.000000E+10 -1.000000E+10 -1.000000E+10 -1.000000E+10  0.000000E+00  0.000000E+00  0.000000E+00  0.000000E+00  0.000000E+00  0.000000E+00  0.000000E+00  0.000000E+00  0.000000E+00  0.000000E+00  0.000000E+00  0.000000E+00       Funtag_D
      47  4.748128E+05  4.289861E+05       45826.6       7.52494     0.0309081      0.524306      0.184438      0.240005    0.00115754   -0.00526707    0.00827811       1.02360       Opttag_D
      47       83.3338       1.12545       1.00000  0.000000E+00       1.00000  0.000000E+00      0.948174       88.9143       1.00000       36.8589  0.000000E+00       8.87196  0.000000E+00       8.87196  0.000000E+00  0.000000E+00      -17.7439       85.2537       3.90356        1       Scatag_D
      47       1931.32       5720.75  0.000000E+00  0.000000E+00       2598.72  0.000000E+00       2626.34       8894.11  0.000000E+00       13260.8       56.2005       10738.4 -1.000000E+10 -1.000000E+10 -1.000000E+10 -1.000000E+10 -1.000000E+10  0.000000E+00  0.000000E+00  0.000000E+00  0.000000E+00  0.000000E+00  0.000000E+00  0.000000E+00  0.000000E+00  0.000000E+00  0.000000E+00  0.000000E+00  0.000000E+00       Funtag_D
      48  4.747977E+05  4.289707E+05       45826.9       9.66502     0.0325212      0.532028      0.184430      0.240006    0.00119683   -0.00538740    0.00827901       1.02374       Opttag_D
      48       83.3232       1.12446       1.00000  0.000000E+00       1.00000  0.000000E+00      0.948180       88.8880       1.00000       36.8303  0.000000E+00       8.87349  0.000000E+00       8.87349  0.000000E+00  0.000000E+00      -17.7470       85.2537       3.90356        1       Scatag_D
      48       1931.74       5722.32  0.000000E+00  0.000000E+00       2598.31  0.000000E+00       2625.75       8895.14  0.000000E+00       13262.9       56.2781       10734.5 -1.000000E+10 -1.000000E+10 -1.000000E+10 -1.000000E+10 -1.000000E+10  0.000000E+00  0.000000E+00  0.000000E+00  0.000000E+00  0.000000E+00  0.000000E+00  0.000000E+00  0.000000E+00  0.000000E+00  0.000000E+00  0.000000E+00  0.000000E+00       Funtag_D
      49  4.747808E+05  4.289598E+05       45821.0       9.36957     0.0338564      0.536917      0.184426      0.240008    0.00122469   -0.00548231    0.00827972       1.02377       Opttag_D
      49       83.3145       1.12368       1.00000  0.000000E+00       1.00000  0.000000E+00      0.948185       88.8645       1.00000       36.8054  0.000000E+00       8.87476  0.000000E+00       8.87476  0.000000E+00  0.000000E+00      -17.7495       85.2537       3.90356        1       Scatag_D
      49       1932.07       5722.64  0.000000E+00  0.000000E+00       2597.86  0.000000E+00       2625.07       8892.83  0.000000E+00       13264.0       56.3438       10730.2 -1.000000E+10 -1.000000E+10 -1.000000E+10 -1.000000E+10 -1.000000E+10  0.000000E+00  0.000000E+00  0.000000E+00  0.000000E+00  0.000000E+00  0.000000E+00  0.000000E+00  0.000000E+00  0.000000E+00  0.000000E+00  0.000000E+00  0.000000E+00       Funtag_D
      50  4.747619E+05  4.289533E+05       45808.6       9.30770     0.0349734      0.539167      0.184424      0.240008    0.00124131   -0.00554709    0.00827873       1.02379       Opttag_D
      50       83.3076       1.12311       1.00000  0.000000E+00       1.00000  0.000000E+00      0.948191       88.8433       1.00000       36.7819  0.000000E+00       8.87583  0.000000E+00       8.87583  0.000000E+00  0.000000E+00      -17.7517       85.2537       3.90356        1       Scatag_D
      50       1931.61       5722.90  0.000000E+00  0.000000E+00       2597.42  0.000000E+00       2624.29       8886.91  0.000000E+00       13264.1       56.3780       10725.0 -1.000000E+10 -1.000000E+10 -1.000000E+10 -1.000000E+10 -1.000000E+10  0.000000E+00  0.000000E+00  0.000000E+00  0.000000E+00  0.000000E+00  0.000000E+00  0.000000E+00  0.000000E+00  0.000000E+00  0.000000E+00  0.000000E+00  0.000000E+00       Funtag_D
      51  4.747413E+05  4.289450E+05       45796.2       10.9139     0.0366350      0.541827      0.184422      0.240010    0.00126244   -0.00564852    0.00827816       1.02384       Opttag_D
      51       83.2979       1.12234       1.00000  0.000000E+00       1.00000  0.000000E+00      0.948199       88.8112       1.00000       36.7469  0.000000E+00       8.87736  0.000000E+00       8.87736  0.000000E+00  0.000000E+00      -17.7547       85.2537       3.90356        1       Scatag_D
      51       1931.34       5723.45  0.000000E+00  0.000000E+00       2596.69  0.000000E+00       2623.31       8881.14  0.000000E+00       13264.3       56.4417       10719.6 -1.000000E+10 -1.000000E+10 -1.000000E+10 -1.000000E+10 -1.000000E+10  0.000000E+00  0.000000E+00  0.000000E+00  0.000000E+00  0.000000E+00  0.000000E+00  0.000000E+00  0.000000E+00  0.000000E+00  0.000000E+00  0.000000E+00  0.000000E+00       Funtag_D
      52  4.747226E+05  4.289419E+05       45780.7       9.79511     0.0378325      0.543492      0.184422      0.240009    0.00127051   -0.00571746    0.00827609       1.02394       Opttag_D
      52       83.2913       1.12188       1.00000  0.000000E+00       1.00000  0.000000E+00      0.948204       88.7891       1.00000       36.7213  0.000000E+00       8.87846  0.000000E+00       8.87846  0.000000E+00  0.000000E+00      -17.7569       85.2537       3.90356        1       Scatag_D
      52       1930.38       5724.56  0.000000E+00  0.000000E+00       2596.07  0.000000E+00       2622.64       8870.42  0.000000E+00       13264.1       56.5200       10716.0 -1.000000E+10 -1.000000E+10 -1.000000E+10 -1.000000E+10 -1.000000E+10  0.000000E+00  0.000000E+00  0.000000E+00  0.000000E+00  0.000000E+00  0.000000E+00  0.000000E+00  0.000000E+00  0.000000E+00  0.000000E+00  0.000000E+00  0.000000E+00       Funtag_D
      53  4.747027E+05  4.289395E+05       45763.2       8.39347     0.0388866      0.545432      0.184420      0.240009    0.00127647   -0.00579269    0.00827356       1.02390       Opttag_D
      53       83.2859       1.12151       1.00000  0.000000E+00       1.00000  0.000000E+00      0.948207       88.7705       1.00000       36.6997  0.000000E+00       8.87935  0.000000E+00       8.87935  0.000000E+00  0.000000E+00      -17.7587       85.2537       3.90356        1       Scatag_D
      53       1929.20       5724.10  0.000000E+00  0.000000E+00       2595.52  0.000000E+00       2621.87       8860.14  0.000000E+00       13264.0       56.5903       10711.7 -1.000000E+10 -1.000000E+10 -1.000000E+10 -1.000000E+10 -1.000000E+10  0.000000E+00  0.000000E+00  0.000000E+00  0.000000E+00  0.000000E+00  0.000000E+00  0.000000E+00  0.000000E+00  0.000000E+00  0.000000E+00  0.000000E+00  0.000000E+00       Funtag_D
      54  4.746775E+05  4.289351E+05       45742.4       11.2434     0.0412832      0.568758      0.184421      0.240012    0.00128780   -0.00598341    0.00827234       1.02393       Opttag_D
      54       83.2739       1.12068       1.00000  0.000000E+00       1.00000  0.000000E+00      0.948215       88.7307       1.00000       36.6551  0.000000E+00       8.88129  0.000000E+00       8.88129  0.000000E+00  0.000000E+00      -17.7626       85.2537       3.90356        1       Scatag_D
      54       1928.63       5724.47  0.000000E+00  0.000000E+00       2594.16  0.000000E+00       2619.89       8850.21  0.000000E+00       13264.7       56.7601       10703.5 -1.000000E+10 -1.000000E+10 -1.000000E+10 -1.000000E+10 -1.000000E+10  0.000000E+00  0.000000E+00  0.000000E+00  0.000000E+00  0.000000E+00  0.000000E+00  0.000000E+00  0.000000E+00  0.000000E+00  0.000000E+00  0.000000E+00  0.000000E+00       Funtag_D
      55  4.746646E+05  4.289306E+05       45734.0       14.6050     0.0439056      0.604020      0.184423      0.240021    0.00129923   -0.00622699    0.00827292       1.02417       Opttag_D
      55       83.2614       1.11974       1.00000  0.000000E+00       1.00000  0.000000E+00      0.948223       88.6879       1.00000       36.6110  0.000000E+00       8.88347  0.000000E+00       8.88347  0.000000E+00  0.000000E+00      -17.7669       85.2537       3.90356        1       Scatag_D
      55       1928.90       5727.08  0.000000E+00  0.000000E+00       2592.50  0.000000E+00       2617.93       8846.86  0.000000E+00       13265.5       56.9102       10698.3 -1.000000E+10 -1.000000E+10 -1.000000E+10 -1.000000E+10 -1.000000E+10  0.000000E+00  0.000000E+00  0.000000E+00  0.000000E+00  0.000000E+00  0.000000E+00  0.000000E+00  0.000000E+00  0.000000E+00  0.000000E+00  0.000000E+00  0.000000E+00       Funtag_D
      56  4.746474E+05  4.289301E+05       45717.3       8.99844     0.0436999      0.600574      0.184422      0.240020    0.00130048   -0.00621462    0.00827187       1.02396       Opttag_D
      56       83.2625       1.11980       1.00000  0.000000E+00       1.00000  0.000000E+00      0.948223       88.6921       1.00000       36.6150  0.000000E+00       8.88322  0.000000E+00       8.88322  0.000000E+00  0.000000E+00      -17.7664       85.2537       3.90356        1       Scatag_D
      56       1928.41       5724.74  0.000000E+00  0.000000E+00       2592.66  0.000000E+00       2617.88       8834.92  0.000000E+00       13265.2       56.8477       10696.6 -1.000000E+10 -1.000000E+10 -1.000000E+10 -1.000000E+10 -1.000000E+10  0.000000E+00  0.000000E+00  0.000000E+00  0.000000E+00  0.000000E+00  0.000000E+00  0.000000E+00  0.000000E+00  0.000000E+00  0.000000E+00  0.000000E+00  0.000000E+00       Funtag_D
      57  4.746326E+05  4.289282E+05       45704.3       7.05605     0.0444443      0.609179      0.184423      0.240023    0.00130519   -0.00629233    0.00827291       1.02391       Opttag_D
      57       83.2591       1.11952       1.00000  0.000000E+00       1.00000  0.000000E+00      0.948225       88.6814       1.00000       36.6038  0.000000E+00       8.88373  0.000000E+00       8.88373  0.000000E+00  0.000000E+00      -17.7675       85.2537       3.90356        1       Scatag_D
      57       1928.89       5724.24  0.000000E+00  0.000000E+00       2592.19  0.000000E+00       2617.30       8826.54  0.000000E+00       13264.9       56.7984       10693.5 -1.000000E+10 -1.000000E+10 -1.000000E+10 -1.000000E+10 -1.000000E+10  0.000000E+00  0.000000E+00  0.000000E+00  0.000000E+00  0.000000E+00  0.000000E+00  0.000000E+00  0.000000E+00  0.000000E+00  0.000000E+00  0.000000E+00  0.000000E+00       Funtag_D
      58  4.746191E+05  4.289270E+05       45692.1       7.64485     0.0460160      0.627779      0.184423      0.240027    0.00130836   -0.00645426    0.00827393       1.02394       Opttag_D
      58       83.2523       1.11903       1.00000  0.000000E+00       1.00000  0.000000E+00      0.948230       88.6584       1.00000       36.5795  0.000000E+00       8.88497  0.000000E+00       8.88497  0.000000E+00  0.000000E+00      -17.7699       85.2537       3.90356        1       Scatag_D
      58       1929.37       5724.56  0.000000E+00  0.000000E+00       2591.14  0.000000E+00       2616.37       8819.60  0.000000E+00       13264.7       56.7821       10689.6 -1.000000E+10 -1.000000E+10 -1.000000E+10 -1.000000E+10 -1.000000E+10  0.000000E+00  0.000000E+00  0.000000E+00  0.000000E+00  0.000000E+00  0.000000E+00  0.000000E+00  0.000000E+00  0.000000E+00  0.000000E+00  0.000000E+00  0.000000E+00       Funtag_D
      59  4.746086E+05  4.289259E+05       45682.7       10.6061     0.0476888      0.646048      0.184428      0.240037    0.00131115   -0.00663655    0.00827602       1.02398       Opttag_D
      59       83.2453       1.11854       1.00000  0.000000E+00       1.00000  0.000000E+00      0.948234       88.6354       1.00000       36.5544  0.000000E+00       8.88618  0.000000E+00       8.88618  0.000000E+00  0.000000E+00      -17.7724       85.2537       3.90356        2       Scatag_D
      59       1930.34       5725.01  0.000000E+00  0.000000E+00       2589.95  0.000000E+00       2615.44       8815.38  0.000000E+00       13263.8       56.7501       10686.0 -1.000000E+10 -1.000000E+10 -1.000000E+10 -1.000000E+10 -1.000000E+10  0.000000E+00  0.000000E+00  0.000000E+00  0.000000E+00  0.000000E+00  0.000000E+00  0.000000E+00  0.000000E+00  0.000000E+00  0.000000E+00  0.000000E+00  0.000000E+00       Funtag_D
      60  4.745931E+05  4.289279E+05       45665.2       7.87093     0.0497371      0.668573      0.184431      0.240051    0.00130605   -0.00686877    0.00827558       1.02397       Opttag_D
      60       83.2371       1.11802       1.00000  0.000000E+00       1.00000  0.000000E+00      0.948238       88.6079       1.00000       36.5246  0.000000E+00       8.88788  0.000000E+00       8.88788  0.000000E+00  0.000000E+00      -17.7758       85.2537       3.90356        1       Scatag_D
      60       1930.14       5724.85  0.000000E+00  0.000000E+00       2588.40  0.000000E+00       2614.38       8804.34  0.000000E+00       13263.1       56.7547       10683.2 -1.000000E+10 -1.000000E+10 -1.000000E+10 -1.000000E+10 -1.000000E+10  0.000000E+00  0.000000E+00  0.000000E+00  0.000000E+00  0.000000E+00  0.000000E+00  0.000000E+00  0.000000E+00  0.000000E+00  0.000000E+00  0.000000E+00  0.000000E+00       Funtag_D
      61  4.745798E+05  4.289302E+05       45649.7       7.06049     0.0512966      0.683662      0.184434      0.240060    0.00130039   -0.00705972    0.00827403       1.02399       Opttag_D
      61       83.2314       1.11770       1.00000  0.000000E+00       1.00000  0.000000E+00      0.948240       88.5901       1.00000       36.5022  0.000000E+00       8.88911  0.000000E+00       8.88911  0.000000E+00  0.000000E+00      -17.7782       85.2537       3.90356        1       Scatag_D
      61       1929.42       5725.07  0.000000E+00  0.000000E+00       2587.15  0.000000E+00       2613.47       8795.95  0.000000E+00       13261.4       56.7555       10680.5 -1.000000E+10 -1.000000E+10 -1.000000E+10 -1.000000E+10 -1.000000E+10  0.000000E+00  0.000000E+00  0.000000E+00  0.000000E+00  0.000000E+00  0.000000E+00  0.000000E+00  0.000000E+00  0.000000E+00  0.000000E+00  0.000000E+00  0.000000E+00       Funtag_D
      62  4.745673E+05  4.289318E+05       45635.5       9.97661     0.0533594      0.700555      0.184438      0.240077    0.00129606   -0.00734446    0.00827287       1.02401       Opttag_D
      62       83.2247       1.11732       1.00000  0.000000E+00       1.00000  0.000000E+00      0.948240       88.5707       1.00000       36.4736  0.000000E+00       8.89078  0.000000E+00       8.89078  0.000000E+00  0.000000E+00      -17.7816       85.2537       3.90356        1       Scatag_D
      62       1928.88       5725.28  0.000000E+00  0.000000E+00       2585.36  0.000000E+00       2612.38       8792.30  0.000000E+00       13257.5       56.7566       10677.0 -1.000000E+10 -1.000000E+10 -1.000000E+10 -1.000000E+10 -1.000000E+10  0.000000E+00  0.000000E+00  0.000000E+00  0.000000E+00  0.000000E+00  0.000000E+00  0.000000E+00  0.000000E+00  0.000000E+00  0.000000E+00  0.000000E+00  0.000000E+00       Funtag_D
      63  4.745542E+05  4.289308E+05       45623.4       8.05055     0.0541388      0.704250      0.184436      0.240082    0.00129866   -0.00746513    0.00827279       1.02393       Opttag_D
      63       83.2227       1.11722       1.00000  0.000000E+00       1.00000  0.000000E+00      0.948239       88.5680       1.00000       36.4630  0.000000E+00       8.89128  0.000000E+00       8.89128  0.000000E+00  0.000000E+00      -17.7826       85.2537       3.90356        1       Scatag_D
      63       1928.84       5724.38  0.000000E+00  0.000000E+00       2584.61  0.000000E+00       2612.00       8786.73  0.000000E+00       13254.2       56.7658       10675.9 -1.000000E+10 -1.000000E+10 -1.000000E+10 -1.000000E+10 -1.000000E+10  0.000000E+00  0.000000E+00  0.000000E+00  0.000000E+00  0.000000E+00  0.000000E+00  0.000000E+00  0.000000E+00  0.000000E+00  0.000000E+00  0.000000E+00  0.000000E+00       Funtag_D
      64  4.745433E+05  4.289265E+05       45616.8       7.95411     0.0542519      0.700625      0.184432      0.240081    0.00130980   -0.00750898    0.00827311       1.02384       Opttag_D
      64       83.2232       1.11722       1.00000  0.000000E+00       1.00000  0.000000E+00      0.948238       88.5741       1.00000       36.4612  0.000000E+00       8.89124  0.000000E+00       8.89124  0.000000E+00  0.000000E+00      -17.7825       85.2537       3.90356        1       Scatag_D
      64       1928.99       5723.41  0.000000E+00  0.000000E+00       2584.39  0.000000E+00       2611.85       8784.98  0.000000E+00       13251.0       56.7755       10675.3 -1.000000E+10 -1.000000E+10 -1.000000E+10 -1.000000E+10 -1.000000E+10  0.000000E+00  0.000000E+00  0.000000E+00  0.000000E+00  0.000000E+00  0.000000E+00  0.000000E+00  0.000000E+00  0.000000E+00  0.000000E+00  0.000000E+00  0.000000E+00       Funtag_D
      65  4.745249E+05  4.289160E+05       45608.9       9.33457     0.0549104      0.695501      0.184425      0.240085    0.00133651   -0.00767024    0.00827696       1.02367       Opttag_D
      65       83.2230       1.11710       1.00000  0.000000E+00       1.00000  0.000000E+00      0.948236       88.5835       1.00000       36.4534  0.000000E+00       8.89165  0.000000E+00       8.89165  0.000000E+00  0.000000E+00      -17.7833       85.2537       3.90356        1       Scatag_D
      65       1930.78       5721.52  0.000000E+00  0.000000E+00       2583.51  0.000000E+00       2611.31       8785.07  0.000000E+00       13243.8       56.8212       10676.0 -1.000000E+10 -1.000000E+10 -1.000000E+10 -1.000000E+10 -1.000000E+10  0.000000E+00  0.000000E+00  0.000000E+00  0.000000E+00  0.000000E+00  0.000000E+00  0.000000E+00  0.000000E+00  0.000000E+00  0.000000E+00  0.000000E+00  0.000000E+00       Funtag_D
      66  4.745133E+05  4.289044E+05       45608.9       14.4056     0.0565179      0.692248      0.184416      0.240097    0.00136600   -0.00797489    0.00828582       1.02349       Opttag_D
      66       83.2207       1.11690       1.00000  0.000000E+00       1.00000  0.000000E+00      0.948230       88.5938       1.00000       36.4358  0.000000E+00       8.89278  0.000000E+00       8.89278  0.000000E+00  0.000000E+00      -17.7856       85.2537       3.90356        1       Scatag_D
      66       1934.92       5719.45  0.000000E+00  0.000000E+00       2581.55  0.000000E+00       2610.35       8792.09  0.000000E+00       13233.9       56.8852       10679.8 -1.000000E+10 -1.000000E+10 -1.000000E+10 -1.000000E+10 -1.000000E+10  0.000000E+00  0.000000E+00  0.000000E+00  0.000000E+00  0.000000E+00  0.000000E+00  0.000000E+00  0.000000E+00  0.000000E+00  0.000000E+00  0.000000E+00  0.000000E+00       Funtag_D
      67  4.744943E+05  4.289021E+05       45592.2       8.69133     0.0568793      0.692521      0.184416      0.240101    0.00137186   -0.00804000    0.00828556       1.02338       Opttag_D
      67       83.2199       1.11681       1.00000  0.000000E+00       1.00000  0.000000E+00      0.948231       88.5930       1.00000       36.4325  0.000000E+00       8.89310  0.000000E+00       8.89310  0.000000E+00  0.000000E+00      -17.7862       85.2537       3.90356        1       Scatag_D
      67       1934.80       5718.25  0.000000E+00  0.000000E+00       2581.14  0.000000E+00       2610.03       8779.74  0.000000E+00       13232.8       56.8621       10678.6 -1.000000E+10 -1.000000E+10 -1.000000E+10 -1.000000E+10 -1.000000E+10  0.000000E+00  0.000000E+00  0.000000E+00  0.000000E+00  0.000000E+00  0.000000E+00  0.000000E+00  0.000000E+00  0.000000E+00  0.000000E+00  0.000000E+00  0.000000E+00       Funtag_D
      68  4.744769E+05  4.288991E+05       45577.8       7.25866     0.0579743      0.708929      0.184414      0.240110    0.00137954   -0.00821408    0.00828668       1.02324       Opttag_D
      68       83.2173       1.11664       1.00000  0.000000E+00       1.00000  0.000000E+00      0.948230       88.5909       1.00000       36.4220  0.000000E+00       8.89406  0.000000E+00       8.89406  0.000000E+00  0.000000E+00      -17.7881       85.2537       3.90356        1       Scatag_D
      68       1935.32       5716.69  0.000000E+00  0.000000E+00       2579.92  0.000000E+00       2609.43       8773.58  0.000000E+00       13230.0       56.7962       10676.1 -1.000000E+10 -1.000000E+10 -1.000000E+10 -1.000000E+10 -1.000000E+10  0.000000E+00  0.000000E+00  0.000000E+00  0.000000E+00  0.000000E+00  0.000000E+00  0.000000E+00  0.000000E+00  0.000000E+00  0.000000E+00  0.000000E+00  0.000000E+00       Funtag_D
      69  4.744630E+05  4.288962E+05       45566.8       7.71691     0.0593773      0.738182      0.184411      0.240119    0.00138713   -0.00843067    0.00828861       1.02306       Opttag_D
      69       83.2146       1.11651       1.00000  0.000000E+00       1.00000  0.000000E+00      0.948226       88.5917       1.00000       36.4091  0.000000E+00       8.89516  0.000000E+00       8.89516  0.000000E+00  0.000000E+00      -17.7903       85.2537       3.90356        1       Scatag_D
      69       1936.22       5714.68  0.000000E+00  0.000000E+00       2578.30  0.000000E+00       2609.08       8771.41  0.000000E+00       13226.1       56.6617       10674.4 -1.000000E+10 -1.000000E+10 -1.000000E+10 -1.000000E+10 -1.000000E+10  0.000000E+00  0.000000E+00  0.000000E+00  0.000000E+00  0.000000E+00  0.000000E+00  0.000000E+00  0.000000E+00  0.000000E+00  0.000000E+00  0.000000E+00  0.000000E+00       Funtag_D
      70  4.744565E+05  4.288931E+05       45563.4       15.1820     0.0618349      0.789787      0.184406      0.240132    0.00139481   -0.00880008    0.00828642       1.02297       Opttag_D
      70       83.2111       1.11647       1.00000  0.000000E+00       1.00000  0.000000E+00      0.948217       88.6006       1.00000       36.3877  0.000000E+00       8.89723  0.000000E+00       8.89723  0.000000E+00  0.000000E+00      -17.7945       85.2537       3.90356        1       Scatag_D
      70       1935.20       5713.72  0.000000E+00  0.000000E+00       2575.33  0.000000E+00       2609.06       8779.23  0.000000E+00       13223.0       56.2425       10671.5 -1.000000E+10 -1.000000E+10 -1.000000E+10 -1.000000E+10 -1.000000E+10  0.000000E+00  0.000000E+00  0.000000E+00  0.000000E+00  0.000000E+00  0.000000E+00  0.000000E+00  0.000000E+00  0.000000E+00  0.000000E+00  0.000000E+00  0.000000E+00       Funtag_D
      71  4.744362E+05  4.288919E+05       45544.3       6.99531     0.0619092      0.791371      0.184406      0.240130    0.00139789   -0.00879513    0.00828489       1.02282       Opttag_D
      71       83.2113       1.11650       1.00000  0.000000E+00       1.00000  0.000000E+00      0.948216       88.6029       1.00000       36.3872  0.000000E+00       8.89714  0.000000E+00       8.89714  0.000000E+00  0.000000E+00      -17.7943       85.2537       3.90356        1       Scatag_D
      71       1934.48       5712.04  0.000000E+00  0.000000E+00       2575.15  0.000000E+00       2609.00       8764.01  0.000000E+00       13222.9       56.1922       10670.5 -1.000000E+10 -1.000000E+10 -1.000000E+10 -1.000000E+10 -1.000000E+10  0.000000E+00  0.000000E+00  0.000000E+00  0.000000E+00  0.000000E+00  0.000000E+00  0.000000E+00  0.000000E+00  0.000000E+00  0.000000E+00  0.000000E+00  0.000000E+00       Funtag_D
      72  4.744273E+05  4.288899E+05       45537.5       5.76795     0.0618892      0.791093      0.184402      0.240124    0.00140317   -0.00877197    0.00828281       1.02281       Opttag_D
      72       83.2121       1.11657       1.00000  0.000000E+00       1.00000  0.000000E+00      0.948215       88.6077       1.00000       36.3872  0.000000E+00       8.89705  0.000000E+00       8.89705  0.000000E+00  0.000000E+00      -17.7941       85.2537       3.90356        1       Scatag_D
      72       1933.51       5711.91  0.000000E+00  0.000000E+00       2574.99  0.000000E+00       2608.86       8760.61  0.000000E+00       13222.4       56.1415       10669.0 -1.000000E+10 -1.000000E+10 -1.000000E+10 -1.000000E+10 -1.000000E+10  0.000000E+00  0.000000E+00  0.000000E+00  0.000000E+00  0.000000E+00  0.000000E+00  0.000000E+00  0.000000E+00  0.000000E+00  0.000000E+00  0.000000E+00  0.000000E+00       Funtag_D
      73  4.744169E+05  4.288850E+05       45531.9       6.64628     0.0625052      0.803026      0.184398      0.240121    0.00141556   -0.00884147    0.00828201       1.02275       Opttag_D
      73       83.2123       1.11662       1.00000  0.000000E+00       1.00000  0.000000E+00      0.948211       88.6169       1.00000       36.3824  0.000000E+00       8.89751  0.000000E+00       8.89751  0.000000E+00  0.000000E+00      -17.7950       85.2537       3.90356        1       Scatag_D
      73       1933.14       5711.26  0.000000E+00  0.000000E+00       2573.94  0.000000E+00       2608.46       8759.78  0.000000E+00       13221.8       56.1186       10667.5 -1.000000E+10 -1.000000E+10 -1.000000E+10 -1.000000E+10 -1.000000E+10  0.000000E+00  0.000000E+00  0.000000E+00  0.000000E+00  0.000000E+00  0.000000E+00  0.000000E+00  0.000000E+00  0.000000E+00  0.000000E+00  0.000000E+00  0.000000E+00       Funtag_D
      74  4.744014E+05  4.288717E+05       45529.7       10.9415     0.0633894      0.816799      0.184387      0.240113    0.00144947   -0.00892435    0.00828461       1.02266       Opttag_D
      74       83.2136       1.11667       1.00000  0.000000E+00       1.00000  0.000000E+00      0.948206       88.6357       1.00000       36.3764  0.000000E+00       8.89837  0.000000E+00       8.89837  0.000000E+00  0.000000E+00      -17.7967       85.2537       3.90356        1       Scatag_D
      74       1934.35       5710.20  0.000000E+00  0.000000E+00       2572.18  0.000000E+00       2606.91       8764.10  0.000000E+00       13220.3       56.4240       10665.2 -1.000000E+10 -1.000000E+10 -1.000000E+10 -1.000000E+10 -1.000000E+10  0.000000E+00  0.000000E+00  0.000000E+00  0.000000E+00  0.000000E+00  0.000000E+00  0.000000E+00  0.000000E+00  0.000000E+00  0.000000E+00  0.000000E+00  0.000000E+00       Funtag_D
      75  4.743849E+05  4.288583E+05       45526.6       9.47704     0.0645944      0.835656      0.184376      0.240105    0.00148367   -0.00905952    0.00828983       1.02260       Opttag_D
      75       83.2146       1.11670       1.00000  0.000000E+00       1.00000  0.000000E+00      0.948203       88.6534       1.00000       36.3694  0.000000E+00       8.89934  0.000000E+00       8.89934  0.000000E+00  0.000000E+00      -17.7987       85.2537       3.90356        1       Scatag_D
      75       1936.79       5709.51  0.000000E+00  0.000000E+00       2570.19  0.000000E+00       2605.73       8762.84  0.000000E+00       13220.1       56.6781       10664.7 -1.000000E+10 -1.000000E+10 -1.000000E+10 -1.000000E+10 -1.000000E+10  0.000000E+00  0.000000E+00  0.000000E+00  0.000000E+00  0.000000E+00  0.000000E+00  0.000000E+00  0.000000E+00  0.000000E+00  0.000000E+00  0.000000E+00  0.000000E+00       Funtag_D
      76  4.743727E+05  4.288520E+05       45520.8       7.71219     0.0648566      0.837906      0.184372      0.240102    0.00149981   -0.00908021    0.00829143       1.02256       Opttag_D
      76       83.2153       1.11667       1.00000  0.000000E+00       1.00000  0.000000E+00      0.948204       88.6584       1.00000       36.3686  0.000000E+00       8.89955  0.000000E+00       8.89955  0.000000E+00  0.000000E+00      -17.7991       85.2537       3.90356        1       Scatag_D
      76       1937.54       5709.12  0.000000E+00  0.000000E+00       2569.74  0.000000E+00       2605.26       8759.35  0.000000E+00       13219.5       56.7843       10663.5 -1.000000E+10 -1.000000E+10 -1.000000E+10 -1.000000E+10 -1.000000E+10  0.000000E+00  0.000000E+00  0.000000E+00  0.000000E+00  0.000000E+00  0.000000E+00  0.000000E+00  0.000000E+00  0.000000E+00  0.000000E+00  0.000000E+00  0.000000E+00       Funtag_D
      77  4.743537E+05  4.288362E+05       45517.5       7.49740     0.0656405      0.845897      0.184361      0.240091    0.00153996   -0.00913261    0.00829641       1.02253       Opttag_D
      77       83.2173       1.11665       1.00000  0.000000E+00       1.00000  0.000000E+00      0.948207       88.6724       1.00000       36.3663  0.000000E+00       8.90000  0.000000E+00       8.90000  0.000000E+00  0.000000E+00      -17.8000       85.2537       3.90356        1       Scatag_D
      77       1939.86       5708.75  0.000000E+00  0.000000E+00       2568.53  0.000000E+00       2604.45       8758.88  0.000000E+00       13218.3       56.9442       10661.7 -1.000000E+10 -1.000000E+10 -1.000000E+10 -1.000000E+10 -1.000000E+10  0.000000E+00  0.000000E+00  0.000000E+00  0.000000E+00  0.000000E+00  0.000000E+00  0.000000E+00  0.000000E+00  0.000000E+00  0.000000E+00  0.000000E+00  0.000000E+00       Funtag_D
      78  4.743471E+05  4.288208E+05       45526.3       12.0860     0.0667998      0.860655      0.184350      0.240079    0.00157945   -0.00921246    0.00830000       1.02250       Opttag_D
      78       83.2200       1.11679       1.00000  0.000000E+00       1.00000  0.000000E+00      0.948209       88.6929       1.00000       36.3629  0.000000E+00       8.90031  0.000000E+00       8.90031  0.000000E+00  0.000000E+00      -17.8006       85.2537       3.90356        1       Scatag_D
      78       1941.55       5708.46  0.000000E+00  0.000000E+00       2567.07  0.000000E+00       2603.76       8768.01  0.000000E+00       13218.6       57.0284       10661.9 -1.000000E+10 -1.000000E+10 -1.000000E+10 -1.000000E+10 -1.000000E+10  0.000000E+00  0.000000E+00  0.000000E+00  0.000000E+00  0.000000E+00  0.000000E+00  0.000000E+00  0.000000E+00  0.000000E+00  0.000000E+00  0.000000E+00  0.000000E+00       Funtag_D
      79  4.743338E+05  4.288211E+05       45512.7       6.72153     0.0667908      0.861249      0.184350      0.240077    0.00157848   -0.00920526    0.00829652       1.02246       Opttag_D
      79       83.2202       1.11680       1.00000  0.000000E+00       1.00000  0.000000E+00      0.948210       88.6926       1.00000       36.3627  0.000000E+00       8.90028  0.000000E+00       8.90028  0.000000E+00  0.000000E+00      -17.8006       85.2537       3.90356        1       Scatag_D
      79       1939.92       5707.97  0.000000E+00  0.000000E+00       2567.13  0.000000E+00       2603.81       8757.33  0.000000E+00       13218.7       56.9662       10660.9 -1.000000E+10 -1.000000E+10 -1.000000E+10 -1.000000E+10 -1.000000E+10  0.000000E+00  0.000000E+00  0.000000E+00  0.000000E+00  0.000000E+00  0.000000E+00  0.000000E+00  0.000000E+00  0.000000E+00  0.000000E+00  0.000000E+00  0.000000E+00       Funtag_D
      80  4.743235E+05  4.288173E+05       45506.1       5.50396     0.0672476      0.869025      0.184346      0.240072    0.00158819   -0.00923394    0.00829287       1.02241       Opttag_D
      80       83.2214       1.11690       1.00000  0.000000E+00       1.00000  0.000000E+00      0.948211       88.6999       1.00000       36.3608  0.000000E+00       8.90036  0.000000E+00       8.90036  0.000000E+00  0.000000E+00      -17.8007       85.2537       3.90356        1       Scatag_D
      80       1938.21       5707.46  0.000000E+00  0.000000E+00       2566.70  0.000000E+00       2603.62       8753.94  0.000000E+00       13219.5       56.9186       10659.8 -1.000000E+10 -1.000000E+10 -1.000000E+10 -1.000000E+10 -1.000000E+10  0.000000E+00  0.000000E+00  0.000000E+00  0.000000E+00  0.000000E+00  0.000000E+00  0.000000E+00  0.000000E+00  0.000000E+00  0.000000E+00  0.000000E+00  0.000000E+00       Funtag_D
      81  4.743156E+05  4.288106E+05       45504.9       5.91610     0.0679015      0.879194      0.184340      0.240065    0.00160529   -0.00928094    0.00829132       1.02240       Opttag_D
      81       83.2232       1.11701       1.00000  0.000000E+00       1.00000  0.000000E+00      0.948213       88.7108       1.00000       36.3587  0.000000E+00       8.90051  0.000000E+00       8.90051  0.000000E+00  0.000000E+00      -17.8010       85.2537       3.90356        1       Scatag_D
      81       1937.49       5707.32  0.000000E+00  0.000000E+00       2566.13  0.000000E+00       2603.22       8754.05  0.000000E+00       13220.6       56.9596       10659.1 -1.000000E+10 -1.000000E+10 -1.000000E+10 -1.000000E+10 -1.000000E+10  0.000000E+00  0.000000E+00  0.000000E+00  0.000000E+00  0.000000E+00  0.000000E+00  0.000000E+00  0.000000E+00  0.000000E+00  0.000000E+00  0.000000E+00  0.000000E+00       Funtag_D
      82  4.742998E+05  4.287931E+05       45506.7       8.17769     0.0692171      0.897760      0.184326      0.240053    0.00164986   -0.00937540    0.00829132       1.02238       Opttag_D
      82       83.2278       1.11718       1.00000  0.000000E+00       1.00000  0.000000E+00      0.948219       88.7337       1.00000       36.3558  0.000000E+00       8.90076  0.000000E+00       8.90076  0.000000E+00  0.000000E+00      -17.8015       85.2537       3.90356        1       Scatag_D
      82       1937.49       5707.13  0.000000E+00  0.000000E+00       2565.27  0.000000E+00       2602.10       8756.41  0.000000E+00       13222.9       57.2172       10658.2 -1.000000E+10 -1.000000E+10 -1.000000E+10 -1.000000E+10 -1.000000E+10  0.000000E+00  0.000000E+00  0.000000E+00  0.000000E+00  0.000000E+00  0.000000E+00  0.000000E+00  0.000000E+00  0.000000E+00  0.000000E+00  0.000000E+00  0.000000E+00       Funtag_D
      83  4.742904E+05  4.287684E+05       45522.0       10.3980     0.0712291      0.923483      0.184303      0.240043    0.00171307   -0.00953520    0.00829328       1.02260       Opttag_D
      83       83.2354       1.11746       1.00000  0.000000E+00       1.00000  0.000000E+00      0.948231       88.7654       1.00000       36.3539  0.000000E+00       8.90110  0.000000E+00       8.90110  0.000000E+00  0.000000E+00      -17.8022       85.2537       3.90356        1       Scatag_D
      83       1938.40       5709.52  0.000000E+00  0.000000E+00       2564.54  0.000000E+00       2601.01       8764.20  0.000000E+00       13227.2       57.6005       10659.5 -1.000000E+10 -1.000000E+10 -1.000000E+10 -1.000000E+10 -1.000000E+10  0.000000E+00  0.000000E+00  0.000000E+00  0.000000E+00  0.000000E+00  0.000000E+00  0.000000E+00  0.000000E+00  0.000000E+00  0.000000E+00  0.000000E+00  0.000000E+00       Funtag_D
      84  4.742775E+05  4.287687E+05       45508.7       5.61968     0.0708713      0.917276      0.184303      0.240042    0.00171220   -0.00949877    0.00829131       1.02253       Opttag_D
      84       83.2350       1.11735       1.00000  0.000000E+00       1.00000  0.000000E+00      0.948232       88.7599       1.00000       36.3548  0.000000E+00       8.90087  0.000000E+00       8.90087  0.000000E+00  0.000000E+00      -17.8017       85.2537       3.90356        1       Scatag_D
      84       1937.48       5708.73  0.000000E+00  0.000000E+00       2564.75  0.000000E+00       2601.06       8755.45  0.000000E+00       13225.3       57.4906       10658.5 -1.000000E+10 -1.000000E+10 -1.000000E+10 -1.000000E+10 -1.000000E+10  0.000000E+00  0.000000E+00  0.000000E+00  0.000000E+00  0.000000E+00  0.000000E+00  0.000000E+00  0.000000E+00  0.000000E+00  0.000000E+00  0.000000E+00  0.000000E+00       Funtag_D
      85  4.742708E+05  4.287657E+05       45505.1       5.37223     0.0708002      0.913983      0.184301      0.240040    0.00171989   -0.00948269    0.00829034       1.02253       Opttag_D
      85       83.2359       1.11729       1.00000  0.000000E+00       1.00000  0.000000E+00      0.948235       88.7581       1.00000       36.3557  0.000000E+00       8.90060  0.000000E+00       8.90060  0.000000E+00  0.000000E+00      -17.8012       85.2537       3.90356        1       Scatag_D
      85       1937.03       5708.81  0.000000E+00  0.000000E+00       2564.91  0.000000E+00       2601.09       8754.17  0.000000E+00       13223.8       57.4043       10657.9 -1.000000E+10 -1.000000E+10 -1.000000E+10 -1.000000E+10 -1.000000E+10  0.000000E+00  0.000000E+00  0.000000E+00  0.000000E+00  0.000000E+00  0.000000E+00  0.000000E+00  0.000000E+00  0.000000E+00  0.000000E+00  0.000000E+00  0.000000E+00       Funtag_D
      86  4.742607E+05  4.287576E+05       45503.1       6.33922     0.0710572      0.911855      0.184293      0.240032    0.00174054   -0.00947414    0.00828794       1.02263       Opttag_D
      86       83.2392       1.11728       1.00000  0.000000E+00       1.00000  0.000000E+00      0.948243       88.7603       1.00000       36.3572  0.000000E+00       8.90013  0.000000E+00       8.90013  0.000000E+00  0.000000E+00      -17.8003       85.2537       3.90356        1       Scatag_D
      86       1935.91       5709.85  0.000000E+00  0.000000E+00       2565.07  0.000000E+00       2601.14       8754.63  0.000000E+00       13221.8       57.3067       10657.4 -1.000000E+10 -1.000000E+10 -1.000000E+10 -1.000000E+10 -1.000000E+10  0.000000E+00  0.000000E+00  0.000000E+00  0.000000E+00  0.000000E+00  0.000000E+00  0.000000E+00  0.000000E+00  0.000000E+00  0.000000E+00  0.000000E+00  0.000000E+00       Funtag_D
      87  4.742524E+05  4.287405E+05       45511.9       11.9981     0.0721520      0.913744      0.184277      0.240015    0.00178415   -0.00949888    0.00828259       1.02293       Opttag_D
      87       83.2477       1.11747       1.00000  0.000000E+00       1.00000  0.000000E+00      0.948262       88.7725       1.00000       36.3603  0.000000E+00       8.89900  0.000000E+00       8.89900  0.000000E+00  0.000000E+00      -17.7980       85.2537       3.90356        1       Scatag_D
      87       1933.41       5713.21  0.000000E+00  0.000000E+00       2565.34  0.000000E+00       2601.02       8764.41  0.000000E+00       13219.5       57.3427       10657.6 -1.000000E+10 -1.000000E+10 -1.000000E+10 -1.000000E+10 -1.000000E+10  0.000000E+00  0.000000E+00  0.000000E+00  0.000000E+00  0.000000E+00  0.000000E+00  0.000000E+00  0.000000E+00  0.000000E+00  0.000000E+00  0.000000E+00  0.000000E+00       Funtag_D
      88  4.742392E+05  4.287379E+05       45501.3       6.55223     0.0724267      0.914313      0.184274      0.240010    0.00179088   -0.00949899    0.00828019       1.02298       Opttag_D
      88       83.2498       1.11754       1.00000  0.000000E+00       1.00000  0.000000E+00      0.948265       88.7755       1.00000       36.3609  0.000000E+00       8.89889  0.000000E+00       8.89889  0.000000E+00  0.000000E+00      -17.7978       85.2537       3.90356        1       Scatag_D
      88       1932.29       5713.86  0.000000E+00  0.000000E+00       2565.41  0.000000E+00       2600.94       8755.25  0.000000E+00       13219.1       57.3654       10657.1 -1.000000E+10 -1.000000E+10 -1.000000E+10 -1.000000E+10 -1.000000E+10  0.000000E+00  0.000000E+00  0.000000E+00  0.000000E+00  0.000000E+00  0.000000E+00  0.000000E+00  0.000000E+00  0.000000E+00  0.000000E+00  0.000000E+00  0.000000E+00       Funtag_D
      89  4.742310E+05  4.287342E+05       45496.8       5.20020     0.0729112      0.916621      0.184270      0.240004    0.00180029   -0.00951271    0.00827691       1.02306       Opttag_D
      89       83.2529       1.11768       1.00000  0.000000E+00       1.00000  0.000000E+00      0.948270       88.7812       1.00000       36.3615  0.000000E+00       8.89871  0.000000E+00       8.89871  0.000000E+00  0.000000E+00      -17.7974       85.2537       3.90356        1       Scatag_D
      89       1930.76       5714.74  0.000000E+00  0.000000E+00       2565.50  0.000000E+00       2600.65       8752.98  0.000000E+00       13218.9       57.4521       10655.9 -1.000000E+10 -1.000000E+10 -1.000000E+10 -1.000000E+10 -1.000000E+10  0.000000E+00  0.000000E+00  0.000000E+00  0.000000E+00  0.000000E+00  0.000000E+00  0.000000E+00  0.000000E+00  0.000000E+00  0.000000E+00  0.000000E+00  0.000000E+00       Funtag_D
      90  4.742242E+05  4.287289E+05       45495.3       5.25113     0.0735041      0.919141      0.184264      0.239996    0.00181374   -0.00953116    0.00827446       1.02316       Opttag_D
      90       83.2570       1.11785       1.00000  0.000000E+00       1.00000  0.000000E+00      0.948276       88.7882       1.00000       36.3625  0.000000E+00       8.89844  0.000000E+00       8.89844  0.000000E+00  0.000000E+00      -17.7969       85.2537       3.90356        1       Scatag_D
      90       1929.62       5715.84  0.000000E+00  0.000000E+00       2565.69  0.000000E+00       2600.40       8752.92  0.000000E+00       13218.6       57.5172       10654.8 -1.000000E+10 -1.000000E+10 -1.000000E+10 -1.000000E+10 -1.000000E+10  0.000000E+00  0.000000E+00  0.000000E+00  0.000000E+00  0.000000E+00  0.000000E+00  0.000000E+00  0.000000E+00  0.000000E+00  0.000000E+00  0.000000E+00  0.000000E+00       Funtag_D
      91  4.742130E+05  4.287157E+05       45497.4       7.95166     0.0745417      0.923038      0.184250      0.239984    0.00184755   -0.00957546    0.00827346       1.02329       Opttag_D
      91       83.2651       1.11801       1.00000  0.000000E+00       1.00000  0.000000E+00      0.948288       88.7978       1.00000       36.3648  0.000000E+00       8.89797  0.000000E+00       8.89797  0.000000E+00  0.000000E+00      -17.7959       85.2537       3.90356        1       Scatag_D
      91       1929.15       5717.31  0.000000E+00  0.000000E+00       2566.28  0.000000E+00       2600.42       8756.84  0.000000E+00       13217.5       57.2486       10652.6 -1.000000E+10 -1.000000E+10 -1.000000E+10 -1.000000E+10 -1.000000E+10  0.000000E+00  0.000000E+00  0.000000E+00  0.000000E+00  0.000000E+00  0.000000E+00  0.000000E+00  0.000000E+00  0.000000E+00  0.000000E+00  0.000000E+00  0.000000E+00       Funtag_D
      92  4.742028E+05  4.287035E+05       45499.3       6.26562     0.0757559      0.927521      0.184237      0.239972    0.00187849   -0.00961988    0.00827424       1.02351       Opttag_D
      92       83.2746       1.11827       1.00000  0.000000E+00       1.00000  0.000000E+00      0.948297       88.8104       1.00000       36.3685  0.000000E+00       8.89749  0.000000E+00       8.89749  0.000000E+00  0.000000E+00      -17.7950       85.2537       3.90356        1       Scatag_D
      92       1929.51       5719.75  0.000000E+00  0.000000E+00       2567.07  0.000000E+00       2600.63       8756.83  0.000000E+00       13216.9       56.9931       10651.6 -1.000000E+10 -1.000000E+10 -1.000000E+10 -1.000000E+10 -1.000000E+10  0.000000E+00  0.000000E+00  0.000000E+00  0.000000E+00  0.000000E+00  0.000000E+00  0.000000E+00  0.000000E+00  0.000000E+00  0.000000E+00  0.000000E+00  0.000000E+00       Funtag_D
      93  4.741964E+05  4.287002E+05       45496.2       4.94526     0.0757857      0.927694      0.184234      0.239973    0.00188694   -0.00961966    0.00827485       1.02348       Opttag_D
      93       83.2756       1.11819       1.00000  0.000000E+00       1.00000  0.000000E+00      0.948298       88.8086       1.00000       36.3686  0.000000E+00       8.89744  0.000000E+00       8.89744  0.000000E+00  0.000000E+00      -17.7949       85.2537       3.90356        1       Scatag_D
      93       1929.80       5719.43  0.000000E+00  0.000000E+00       2567.18  0.000000E+00       2600.69       8755.16  0.000000E+00       13216.1       56.8485       10650.9 -1.000000E+10 -1.000000E+10 -1.000000E+10 -1.000000E+10 -1.000000E+10  0.000000E+00  0.000000E+00  0.000000E+00  0.000000E+00  0.000000E+00  0.000000E+00  0.000000E+00  0.000000E+00  0.000000E+00  0.000000E+00  0.000000E+00  0.000000E+00       Funtag_D
      94  4.741863E+05  4.286920E+05       45494.3       5.46388     0.0759753      0.928963      0.184228      0.239974    0.00190788   -0.00961617    0.00827609       1.02348       Opttag_D
      94       83.2789       1.11804       1.00000  0.000000E+00       1.00000  0.000000E+00      0.948297       88.8052       1.00000       36.3690  0.000000E+00       8.89728  0.000000E+00       8.89728  0.000000E+00  0.000000E+00      -17.7946       85.2537       3.90356        1       Scatag_D
      94       1930.38       5719.40  0.000000E+00  0.000000E+00       2567.52  0.000000E+00       2601.02       8755.60  0.000000E+00       13214.3       56.5149       10649.6 -1.000000E+10 -1.000000E+10 -1.000000E+10 -1.000000E+10 -1.000000E+10  0.000000E+00  0.000000E+00  0.000000E+00  0.000000E+00  0.000000E+00  0.000000E+00  0.000000E+00  0.000000E+00  0.000000E+00  0.000000E+00  0.000000E+00  0.000000E+00       Funtag_D
      95  4.741791E+05  4.286814E+05       45497.7       7.60046     0.0765374      0.932021      0.184219      0.239967    0.00193497   -0.00960470    0.00827649       1.02355       Opttag_D
      95       83.2852       1.11798       1.00000  0.000000E+00       1.00000  0.000000E+00      0.948299       88.8042       1.00000       36.3705  0.000000E+00       8.89704  0.000000E+00       8.89704  0.000000E+00  0.000000E+00      -17.7941       85.2537       3.90356        1       Scatag_D
      95       1930.56       5720.17  0.000000E+00  0.000000E+00       2568.07  0.000000E+00       2601.94       8759.58  0.000000E+00       13212.7       56.2103       10648.4 -1.000000E+10 -1.000000E+10 -1.000000E+10 -1.000000E+10 -1.000000E+10  0.000000E+00  0.000000E+00  0.000000E+00  0.000000E+00  0.000000E+00  0.000000E+00  0.000000E+00  0.000000E+00  0.000000E+00  0.000000E+00  0.000000E+00  0.000000E+00       Funtag_D
      96  4.741695E+05  4.286754E+05       45494.1       5.81218     0.0769659      0.937365      0.184214      0.239961    0.00195024   -0.00959593    0.00827474       1.02358       Opttag_D
      96       83.2897       1.11799       1.00000  0.000000E+00       1.00000  0.000000E+00      0.948298       88.8040       1.00000       36.3713  0.000000E+00       8.89672  0.000000E+00       8.89672  0.000000E+00  0.000000E+00      -17.7934       85.2537       3.90356        1       Scatag_D
      96       1929.75       5720.48  0.000000E+00  0.000000E+00       2568.45  0.000000E+00       2602.85       8757.20  0.000000E+00       13211.9       56.1145       10647.3 -1.000000E+10 -1.000000E+10 -1.000000E+10 -1.000000E+10 -1.000000E+10  0.000000E+00  0.000000E+00  0.000000E+00  0.000000E+00  0.000000E+00  0.000000E+00  0.000000E+00  0.000000E+00  0.000000E+00  0.000000E+00  0.000000E+00  0.000000E+00       Funtag_D
      97  4.741574E+05  4.286668E+05       45490.6       5.58711     0.0777473      0.948922      0.184207      0.239949    0.00197205   -0.00957603    0.00826969       1.02358       Opttag_D
      97       83.2974       1.11807       1.00000  0.000000E+00       1.00000  0.000000E+00      0.948297       88.8048       1.00000       36.3727  0.000000E+00       8.89613  0.000000E+00       8.89613  0.000000E+00  0.000000E+00      -17.7923       85.2537       3.90356        1       Scatag_D
      97       1927.39       5720.52  0.000000E+00  0.000000E+00       2569.07  0.000000E+00       2604.82       8756.45  0.000000E+00       13211.3       56.1392       10645.0 -1.000000E+10 -1.000000E+10 -1.000000E+10 -1.000000E+10 -1.000000E+10  0.000000E+00  0.000000E+00  0.000000E+00  0.000000E+00  0.000000E+00  0.000000E+00  0.000000E+00  0.000000E+00  0.000000E+00  0.000000E+00  0.000000E+00  0.000000E+00       Funtag_D
      98  4.741503E+05  4.286575E+05       45492.7       7.45828     0.0784660      0.958972      0.184201      0.239940    0.00199578   -0.00957186    0.00826677       1.02356       Opttag_D
      98       83.3044       1.11809       1.00000  0.000000E+00       1.00000  0.000000E+00      0.948296       88.8045       1.00000       36.3731  0.000000E+00       8.89567  0.000000E+00       8.89567  0.000000E+00  0.000000E+00      -17.7913       85.2537       3.90356        1       Scatag_D
      98       1926.03       5720.27  0.000000E+00  0.000000E+00       2569.65  0.000000E+00       2607.15       8759.65  0.000000E+00       13211.3       56.1360       10642.5 -1.000000E+10 -1.000000E+10 -1.000000E+10 -1.000000E+10 -1.000000E+10  0.000000E+00  0.000000E+00  0.000000E+00  0.000000E+00  0.000000E+00  0.000000E+00  0.000000E+00  0.000000E+00  0.000000E+00  0.000000E+00  0.000000E+00  0.000000E+00       Funtag_D
      99  4.741416E+05  4.286504E+05       45491.3       5.25237     0.0789612      0.965485      0.184196      0.239936    0.00201403   -0.00958288    0.00826618       1.02351       Opttag_D
      99       83.3092       1.11807       1.00000  0.000000E+00       1.00000  0.000000E+00      0.948294       88.8041       1.00000       36.3731  0.000000E+00       8.89538  0.000000E+00       8.89538  0.000000E+00  0.000000E+00      -17.7908       85.2537       3.90356        1       Scatag_D
      99       1925.76       5719.70  0.000000E+00  0.000000E+00       2570.06  0.000000E+00       2608.99       8758.28  0.000000E+00       13211.7       56.0893       10640.7 -1.000000E+10 -1.000000E+10 -1.000000E+10 -1.000000E+10 -1.000000E+10  0.000000E+00  0.000000E+00  0.000000E+00  0.000000E+00  0.000000E+00  0.000000E+00  0.000000E+00  0.000000E+00  0.000000E+00  0.000000E+00  0.000000E+00  0.000000E+00       Funtag_D

   MaxLik Scaling Cycle 0108

   Wilson plot

   Statistics in resolution bins after refinement cycle 0003
```


|  |  |
| --- | --- |
|  | - Rxpct factors plot- Rxpct factors table- <Log-Lik Gain>- Ln Sigma A- Correlation Coefficients |


```
   Iters     Tot_Funct    Xray_Funct    Geom_Funct          Grms     rmsD2init     maxD2init             R         Rfree           LLG       LLGfree     rms(bond)    rms(angle)
   Iters             K   B_IMPF_FRAG        K_MISS   B_TEMP_MISS   K_IMPF_MISS   B_IMPF_MISS        K_SOLV   B_TEMP_SOLV   K_IMPF_SOLV   B_IMPF_SOLV        B_TEMP     B_TEMP_11     B_TEMP_12     B_TEMP_22     B_TEMP_13     B_TEMP_23     B_TEMP_33      K_WILSON      B_WILSON      FGCYCLES
   Iters          BOND         ANGLE      IMPROPER       TORSION    SINTORSION      TRIGONAL        PLANAR  BCORRELATION       CONTACT     IDEALDIST        CHIRAL    SIMILARITY       OCCSSUM  UTILDISTANCE     UTILANGLE   UTILTORSION        RHOFIT        ADP-01        ADP-02        ADP-03        ADP-04        ADP-05        ADP-06        ADP-07        ADP-08        ADP-09        ADP-10      RHOFITPC            QM
     100  4.741326E+05  4.286404E+05       45492.2       4.59577     0.0794614      0.976495      0.184190      0.239935    0.00203968   -0.00961111    0.00826774       1.02341       Opttag_D
     100       83.3140       1.11796       1.00000  0.000000E+00       1.00000  0.000000E+00      0.948293       88.8021       1.00000       36.3720  0.000000E+00       8.89508  0.000000E+00       8.89508  0.000000E+00  0.000000E+00      -17.7902       85.2537       3.90356        1       Scatag_D
     100       1926.48       5718.61  0.000000E+00  0.000000E+00       2570.51  0.000000E+00       2611.06       8758.98  0.000000E+00       13212.5       55.9291       10638.2 -1.000000E+10 -1.000000E+10 -1.000000E+10 -1.000000E+10 -1.000000E+10  0.000000E+00  0.000000E+00  0.000000E+00  0.000000E+00  0.000000E+00  0.000000E+00  0.000000E+00  0.000000E+00  0.000000E+00  0.000000E+00  0.000000E+00  0.000000E+00       Funtag_D
   Geometry Restraint Deviations SCREEN Information

 
 No -bcorrel_zeroocchydrogens_factor flag found - keeping default: will set B for OCC=0 hydrogens to    1.200 times heavy-atom B


 TLS information. Total number of groups:     2


 Group   Natoms    FixRB   FixTLS   Group Tag    Group Spec
     1     2781      Y        Y     tls1    { A|* }
     2     2770      Y        Y     tls2    { B|* }
 
NOTE BUSTER_TLS_SET tls1 { A|* }
NOTE BUSTER_TLS_T   tls1     -9.84122E-02  2.63375E-02  4.22310E-02 -5.87256E-02  1.84806E-02 -4.63797E-02
NOTE BUSTER_TLS_L   tls1      2.53184E+00  2.05865E+00  1.86779E+00 -8.79892E-01 -1.59062E+00  7.18718E-01
NOTE BUSTER_TLS_S   tls1     -1.17821E-02 -3.86196E-01  5.27491E-01 -4.35968E-01  2.67147E-01 -2.69276E-01  6.09961E-02 -3.35657E-01
NOTE BUSTER_TLS_O   tls1     -2.30832E+01 -8.41146E+01  1.12764E+01
NOTE BUSTER_TLS_FIX tls1 ALL
 
NOTE BUSTER_TLS_SET tls2 { B|* }
NOTE BUSTER_TLS_T   tls2     -1.73365E-01 -1.10956E-01  1.17182E-01  1.49139E-02  2.71700E-02 -2.71783E-02
NOTE BUSTER_TLS_L   tls2      1.57079E+00  1.30451E+00  1.13623E+00  8.10314E-01 -1.11889E+00 -8.20951E-01
NOTE BUSTER_TLS_S   tls2     -7.19371E-02  1.20184E-01 -9.00838E-02  1.24667E-02  1.65761E-01  7.66243E-02 -1.29102E-01  7.22209E-02
NOTE BUSTER_TLS_O   tls2      2.22817E+01 -7.23258E+01  3.51644E+00
NOTE BUSTER_TLS_FIX tls2 ALL

 For final position, Geometry function summary
     term                           number     weight       rms        GooF      function     fn/numb
     ================================================================================================
     BOND bond lengths (angs)       5665       2.000      0.0083       0.412      1926.48       0.340
     ANGL bond angles (degs)        7687       2.000        1.02       0.610      5718.61       0.744
     TORS split-harmonic tors       1275       0.000       18.58       1.168         0.00       0.000
     SINTOR sinusoidal tors         1275       2.000       18.58                  2570.51       2.016
     PLAN general plane (angs)       967       5.000      0.0147       0.735      2611.06       2.700
     BCORrelations (angs^2)         5665      10.000       1.102       0.393      8758.98       1.546
     CONT (bad contacts)               0       5.000                                 0.00
     IDEAL(ideal-dist contact)      5771       4.000                             13212.47
     CHIRAL (gelly semiharmon)       740       5.000                                55.93
     SIM similarity restraint for NCS and/or TARGET                              10638.20
     OCCSUM restraint on sum of occupanies                                           0.00       as none defined
     DISTAN utility restraints interatomic distance                                  0.00       as none defined
     UTILANGLE utility angle restraints                                              0.00       as none defined
     UTILTOR utility restraints on torsion angles                                    0.00       as none defined
     Peptide omega torsion angles                           2.96 degs - monitor only

 Weighted rms      0.0083         1.023  (temporary rms bond and angle for BUSTER to pick up!)
 Weighted intra-residual rms(bond) for StdProtein (excluding H)      0.0081
 Weighted intra-residual rms(bond) for Buffer     (excluding H)      0.0037
 Outputing geometry progress graph in plotmtv format to file:Geom_Res.mtv

 ############### Running final geometry/screen analysis (screen_final.txt)

 s/r gelly normal termination
```

Statistics and scale factors vs. cycle number
```

|  |  |  |
| --- | --- | --- |
|  | - Rxpct factors- <Log-Lik Gain>- GEOM Residuals- Ln Sigma_A_ | - Scale Factors- B Factors- Imperfection Factors |

```
 Writing F_calc(model+solvent) to complete.mtz

   Final Geometry Information


---


### 2. Final results

Statistics and scale factors vs. cycle number
```

|  |  |  |
| --- | --- | --- |
|  | - Rxpct factors- <Log-Lik Gain>- GEOM Residuals- Ln Sigma_A_ | - Scale Factors- B Factors- Imperfection Factors |

```
   Average Figure of Merit
   Structure Factor Amplitudes


 # normal termination

  Program stopped.
```

closesok message: time spent in tree = 1703.34 (user) 33.29 (sys)

---

# Normal termination

```
 Resources usage summary:
     Time spent in client ...................  1703.35 (user)       33.31 (system)
     Time spent in scripts (    27 calls) ...     2.26 (user)        0.15 (system)
     Time spent in server ...................     0.00 (user)        0.00 (system)

     Max resident memory used (kb) .......... 382920
     Number of page faults .................. 0 (major)    1009278 (minor)
     Number of context switches ............. 2738 (niced)    6621 (other)
```
